# Morphological Traits Are Not Consistently Related to Population Size in Four Migratory Caribou Populations Across North America

**DOI:** 10.1002/ece3.70468

**Published:** 2024-10-15

**Authors:** Barbara Vuillaume, Mathieu Leblond, Marco Festa‐Bianchet, Steeve D. Côté

**Affiliations:** ^1^ Science and Technology Branch Environment and Climate Change Canada Ottawa Canada; ^2^ Département de Biologie Université de Sherbrooke Sherbrooke Canada; ^3^ Département de Biologie Caribou Ungava and Centre d'études Nordiques, Université Laval Quebec City Canada

**Keywords:** aerial survey, large herbivores, morphological traits, population dynamics, *Rangifer tarandus*, wildlife management

## Abstract

To develop effective management to maintain or restore populations of large herbivores, wildlife managers require sound empirical data on their variations in size and associated parameters. Many studies have highlighted links between morphological traits of individuals and population density; however, less attention has been devoted to whether or not morphological traits can reliably inform on population size in years when no population estimates are available. We evaluated the relationships between three morphological traits (hind foot length, body mass, and body fat) and population size interpolated over three decades, for four migratory caribou (*Rangifer tarandus*) herds in northern Canada and Alaska. Our sample included 8865 measurements of 4473 individuals. We used a Bayesian modeling approach to evaluate the relationships between morphology and population size across different sex and age classes, considering different temporal scales and, when possible, phases of population growth or decline. We found that morphological traits were not consistently linked to population size. Statistically significant relationships existed for some combinations of herd and age classes, but weak to absent relationships were more common. Our study suggests that morphological traits alone cannot replace data obtained from aerial surveys to approximate population size when population trends are unknown. We discuss the usefulness of morphological traits to explain population size, and recognize their role as complementary metrics to inform the management and conservation of large herbivores, but conclude that morphological data should not be used to predict population size without information on population trends.

## Graphical abstract

We tested the relationships between three morphological traits and population size in four *Rangifer tarandus* herds in northern Canada and Alaska. Based on 8865 measurements of 4473 individuals over four decades, we conclude that without prior information on population trends, morphological data in most cases should not be used to predict population size.

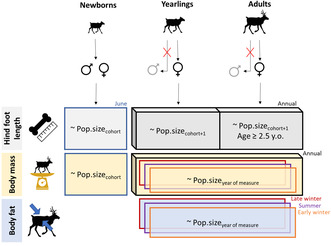

## 1 Introduction

Wildlife managers aim to maintain or restore the size of animal populations that have key ecological roles, or provide important socio‐economic values such as harvests for recreation or subsistence (Haight and Gobster 2009; Kideghesho, Rija 2019). To achieve these goals, wildlife managers require sound empirical data on temporal changes in population size (Frederiksen et al. 2014; Koons et al. 2015). For large herbivores, these data are often obtained using aerial surveys (Pettorelli et al. 2007). Aerial surveys can impose high financial and logistical expenses, especially for species ranging across broad or remote areas (Morellet et al. 2007; Duquette et al. 2015). Consequently, gaps between surveys often span several years (Hauser, Pople, and Possingham 2006). Moreover, unfavorable meteorological conditions impeding aircraft flight (Redfern et al. 2002), reduced visibility of animals under closed canopy cover (Mourão et al. 1994; Morellet et al. 2007), or sparse distribution of animals causing some individuals to be missed (Mourão et al. 1994) are among the many confounding factors that can lead to imprecise population estimates from aerial surveys. Because of these limits, wildlife managers have historically used complementary information to gain knowledge about large herbivore populations in years when aerial survey data are not available. These metrics include demographic parameters such as estimates of survival or reproduction based on composition surveys (Sibly and Hone 2002; Riecke et al. 2019), hunting statistics such as harvest rate or harvest per unit effort (Ueno et al. 2014; Fukasawa, Osada, and Iijima 2020), or indirect ecological indicators (Morellet et al. 2007) including morphological traits (Zannèse et al. 2006; Couturier et al. 2010; Monteith et al. 2014).

The hypothesis that the morphological traits of large herbivores may be indirect indicators of abundance lies on the reciprocal relationship between individual morphology and population dynamics. On one hand, individual traits such as body size or condition, can influence survival and reproduction, and consequently affect population growth (Mahoney and Schaefer 2002; Millán et al. 2022). For example, mothers in poor condition may produce fewer offspring (Hewison and Gaillard 2001) of smaller size (Côté and Festa‐Bianchet 2001), whose fitness may be reduced (Festa‐Bianchet, Jorgenson, and Réale 2000; Steinheim et al. 2002). On the other hand, population density may, in turn, influence individual condition. As density increases, intra‐specific competition may reduce per capita energy intake (Mahoney and Schaefer 2002; Côté et al. 2004). Reduced energy intake in adult females may lead to compromises in resource allocation; ranging from mothers producing a lower‐quality milk, to females skipping a reproductive opportunity (Landete‐Castillejos et al. 2003; Oates et al. 2021). High population density can thus lead to reduced recruitment and juvenile growth (Clutton‐Brock, Albon, and Guinness 1987; Coulson et al. 2001). When individuals are not limited by intra‐specific competition, they can grow rapidly. Mothers in good condition usually produce larger offspring with high survival (Taillon et al. 2012; Tveraa et al. 2013; Lamb et al. 2023). Consequently, as large herbivore populations fluctuate in size, so does the condition of individuals (Morellet et al. 2007). In northern environments, where the timing of access to high‐quality forage is restricted, these relationships can be particularly strong, especially for capital breeders that rely on body reserves to reproduce and survive the winter (Harding et al. 2011; Desforges et al. 2021; Saalfeld et al. 2021).

Density‐dependent variations in the morphological traits of large herbivores are well documented. The mass of juvenile roe deer (*Capreolus capreolus*, Toïgo et al. 2006), moose (*Alces alces*, Ferguson, Bisset, and Messier 2000), and caribou (called reindeer in Eurasia, *Rangifer tarandus*, Couturier et al. 2009), hind foot length of juvenile roe deer (Zannèse et al. 2006), and adult body size in roe deer (Douhard et al. 2013) and red deer (*Cervus elaphus*, Mysterud et al. 2001) were shown to decrease as population size increased. Similarly, when populations were reduced or declined naturally, the birth mass of reindeer (Skogland 1990), roe deer (Hewison et al. 2002), and white‐tailed deer (*Odocoileus virginianus*, Ashley, McCullough, and Robinson 1998) were shown to increase. Juveniles are particularly sensitive to density‐dependent effects because intra‐specific competition and limited resources may constrain their development from the fetal stage to weaning, with potential carry‐over effects that can persist throughout their entire life (Parker, Barboza, and Gillingham 2009; Garel et al. 2011; Scornavacca et al. 2016). In adult *Rangifer*, the relationship between morphological traits and changes in population size is tenuous. For capital breeders such as caribou, behavioral and physiological adaptations can mask density‐dependent effects up to a threshold, reducing the strength of these effects on adult mass, and repercussions on population size (Barboza, Shively, and Thompson 2024). Adult female caribou can adjust their levels of ingestion and physical activity according to the availability and quality of resources. By conserving protein, they can meet the energy requirements of reproduction even when high population density restricts access to resources and body mass is reduced (Barboza et al. 2020; Barboza, Shively, and Thompson 2024). Migratory *Rangifer*, compared to other large herbivores, may also mitigate density‐dependent effects by adjusting their space use, habitat selection, or social behaviors (Teitelbaum et al. 2015; Le Corre, Dussault, and Côté 2020; Webber et al. 2024). The relationship between individual condition and population size may also vary with demographic phase of growth and decline, especially in populations showing large fluctuations in abundance (Bowyer et al. 2014; Gunn 2003).

**FIGURE 1:**
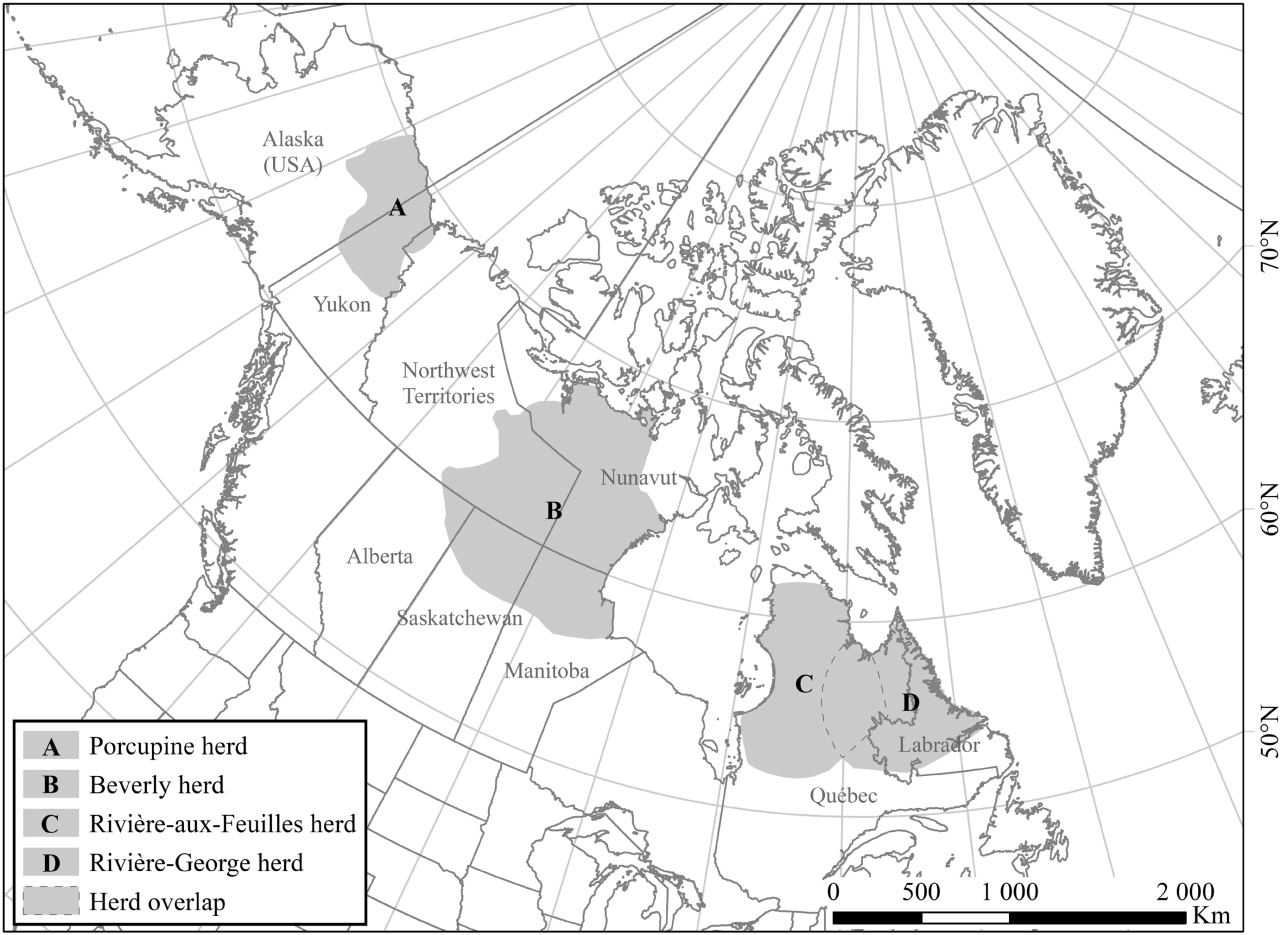
Migratory caribou (*Rangifer tarandus*) herds monitored in this study: (A) the distribution (2005–2016) of the Porcupine herd (*source* = http://www.pcmb.ca, accessed 15 Jun 2023), (B) the distribution (pre‐1994) of the Beverly herd, which overlaps the distributions of the Ahiak, Bathurst, and Qamanirjuaq herds (*source* = http://arctic‐caribou.com, accessed 15 Jun 2023), (C) the distribution (1991–2010) of the Rivière‐aux‐Feuilles herd (data source = http://www.caribou‐ungava.ulaval.ca, accessed 15 Jun 2023), and (D) the distribution (1986–2010) of the Rivière‐George herd (data source = same as C).

Wildlife managers often record the morphological traits of large herbivores, either to identify potential determinants of demographic parameters, assess density‐dependence effects, or for other purposes such as assessments of individual health. As body condition and other morphological traits may have both immediate and chronic responses to density‐dependent processes and can affect both survival and reproduction, it appears important to investigate the reliability of morphological traits to inform on population size. To our knowledge, the question of whether or not morphological traits are reliable complementary indicators of population size in large herbivores remains unanswered. Yet, some studies tried to predict demographic rates in plants and ungulates based on the relationships between functional traits and demographic performance (Easterling, Ellner, and Dixon 2000; Traill et al. 2021). We tackled this question by evaluating the relationships between hind foot length, body mass, or body fat of individuals and population size in a capital breeder, migratory caribou. We had access to large datasets spanning several decades for four migratory caribou herds distributed across northern Canada and Alaska: the Porcupine, Beverly, Rivière‐aux‐Feuilles, and Rivière‐George herds (Figure 1). Our analyses used a Bayesian modeling approach, with uninformative priors. We aimed to determine if any morphological traits could predict migratory caribou population size with an accuracy adequate to inform management.

The three morphological traits we considered were previously identified as appropriate indices of body condition in migratory caribou (Ezenwa, Jolles, and O'Brien 2009; Taillon et al. 2011; Flores‐Saavedra et al. 2018). Because caribou leg bones grow rapidly during the first 2 years then stop growing (Parker 1981), we evaluated the usefulness of hind foot length to assess population trends retrospectively. We predicted that hind foot length would reflect population size during the year following birth, with individuals born at low density being taller than individuals born at high density regardless of current density (Hewison et al. 2002). Contrary to skeletal size, body mass and body fat should be more representative of current population size, thus we predicted that they would decrease with increasing population size in the year of measurement. We evaluated this prediction annually for mass, as well as seasonally for mass and fat to consider intra‐annual variations in these traits (Couturier et al. 2009; Parker, Barboza, and Gillingham 2009). We also accounted for other factors known to affect population dynamics in caribou, such as year of measurement, cohort year and season (to consider annual and seasonal variations in resource availability), phases of population growth and decline (Bonenfant et al. 2009), reproductive status of adult females, and sex and age class of measured individuals. By using data from four herds spread over a continent and across several decades, we aimed to characterize these relationships in a wide variety of contexts so that our results could have broad applicability.

Many additional extrinsic factors not considered in this study are known to influence population size and morphological traits in large herbivores. For example, habitat quality and weather can influence the population dynamics (Priadka et al. 2022), body size (Vannini et al. 2021), and body mass of large herbivores (Toïgo et al. 2006; Giroux et al. 2014). In addition, predation pressure (McLellan et al. 2012; Grange et al. 2015), parasites (Simard et al. 2016; Coulson et al. 2018), biting insects (Raponi et al. 2018; Benedict and Barboza 2022), and human activities (Schaefer 2003; Buuveibaatar et al. 2016) can all influence individual life‐histories of herbivores and their population dynamics. Most of these external influences were of unknown magnitude and likely varied extensively across the broad spatial and temporal scales of our study. We therefore focused on the relationship between morphological traits and population size to track population trends in the absence of data on other external factors, a context often representative of management and conservation of migratory caribou in North America.

## 2 Material and Methods

### 2.1 Migratory Caribou Populations and Distribution Ranges

Our study combines data from four migratory caribou herds (Figure 1). Data were collected over the following periods: Porcupine: 1987–1998; Beverly: 1980–1987; Rivières‐aux‐Feuilles: 1988–2010; Rivière‐George: 1978–2010. Caribou from the Porcupine herd are from the *R. t. granti* sub‐species and are part of the barren‐ground Designatable Unit (DU, *sensu* COSEWIC 2011). Their distribution range extended over 250,000 km^2^ across Alaska, the Yukon and the Northwest Territories (Chan‐McLeod, White, and Russell 1999; Russell and McNeil 2005). Caribou from the Beverly herd are from the *R. t. groenlandicus* sub‐species and are also in the barren‐ground DU. Their distribution range covered 700,000 km^2^ in Nunavut, the Northwest Territories and northern Alberta, Saskatchewan and Manitoba (Klein et al. 1999; Adamczewski et al. 2014). Following a steep population decline during the late 1990s, their distribution retracted and shifted northward (Nagy et al. 2011; Adamczewski et al. 2015). This herd may no longer exist as a distinct population (Adamczewski et al. 2015). In northern Québec and Labrador, the Rivière‐aux‐Feuilles (RAF) and Rivière‐George (RG) herds are part of the *R. t. caribou* sub‐species and eastern migratory DU (COSEWIC 2011). These two herds have shown large fluctuations in abundance accompanied by changes in distribution (Taillon, Brodeur, and Rivard 2016; Le Corre, Dussault, and Côté 2020). At their peak, the two ranges covered 1.2 M km^2^ and overlapped in winter (COSEWIC 2017). Following a sharp decline in abundance—especially the RG which declined by 99%—these herds became spatially segregated in 2012 (Le Corre, Dussault, and Côté 2020).

### 2.2 Population Size—Raw and Interpolated Data

Population monitoring data existed for the four migratory caribou herds, however the methodological approach, the frequency of surveys, and the precision of the ensuing data varied greatly among herds and over time. Several surveys had a wide confidence interval (CI) around estimates or did not have any assessment of uncertainty (Table A1.1). Globally, surveys conducted prior to the early 1980s were based on observations of breeding females on calving grounds or classification of all animals observed during fall migrations. These surveys likely underestimated herd size (Couturier et al. 1990) and lacked error estimates. Beginning in the mid‐1980s, visual surveys were gradually replaced by the interpretation of photographs taken during aerial surveys (Russell et al. 1996).

**FIGURE 2:**
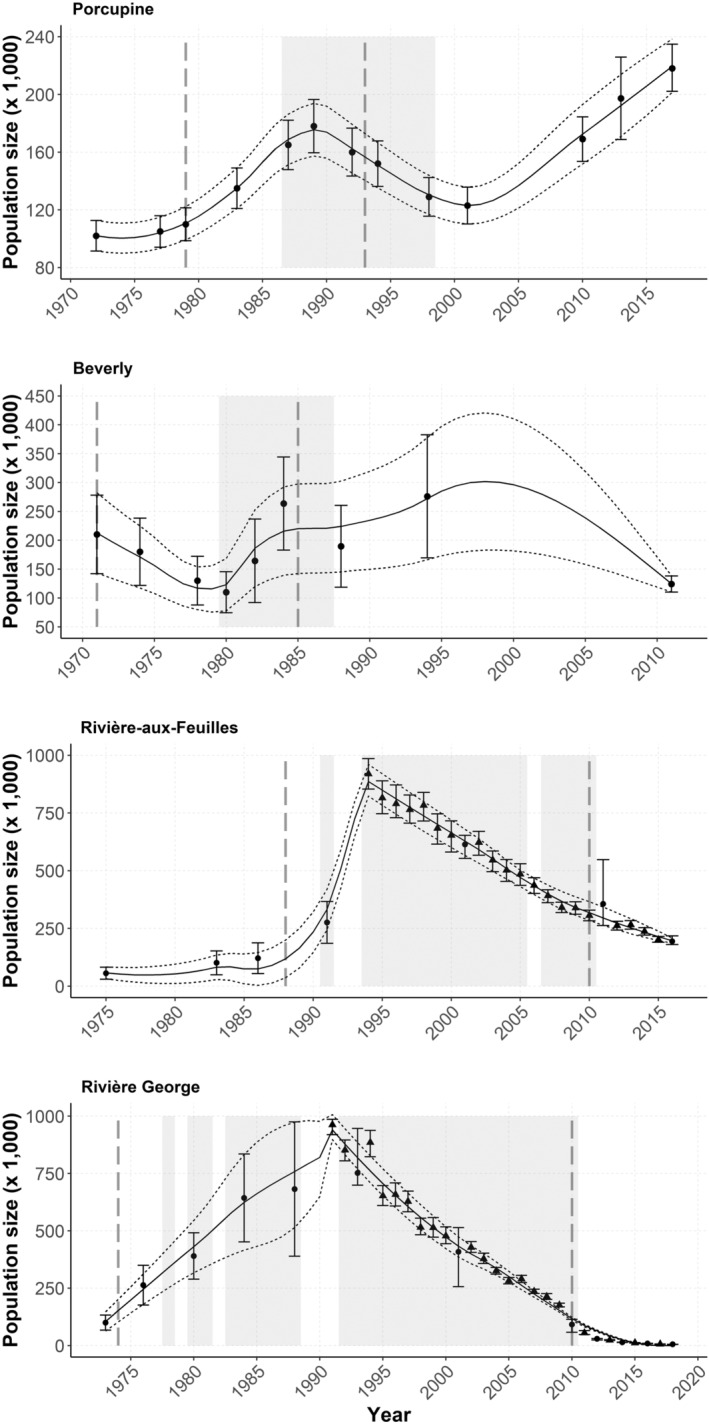
Population estimates of the Porcupine, Beverly, Rivière‐aux‐Feuilles, and Rivière‐George migratory caribou (*Rangifer tarandus*) herds. Population estimates were obtained using aerial surveys (dots) and Integrated Population Model estimates (triangles; Rivière‐aux‐Feuilles and Rivière‐George only), all presented with their 95% confidence intervals (see Appendix S1 for details). We generated population trajectories using loess smoothing. Shaded areas show periods when morphological traits were measured, and dotted vertical lines delimit cohorts for which morphological traits were measured.

The Porcupine herd was first estimated at 102,000 individuals in 1972 based on a field count (Urquhart 1983). Thereafter, 12 aerial surveys were performed between 1977 and 2017 (see Table A1.1 for a complete description of survey data and references). During this period, the herd peaked twice (Figure 2), once in 1989 at an estimated 178,000 individuals (Arthur et al. 2003) and again in 2017 at 218,000 individuals ±15,894 (95% CI; Porcupine Caribou Technical Committee 2021). The Beverly herd was monitored as a distinct population from 1971 to 2011, when nine surveys revealed a first decline from an estimated 210,000 individuals in 1971 to 110,000 in 1980 (Gunn and Decker 1982). The herd reached its highest known peak in 1994 at about 276,000 ± 106,600 (95% CI; Williams 1995), before declining again to 124,200 ± 14,000 (95% CI) in 2011 (Campbell et al. 2012). We excluded Beverly estimates obtained after 2011 because they may have encompassed individuals from the Ahiak herd (COSEWIC 2016). The RAF and RG herds were respectively estimated at around 56,000 individuals in 1975 (Le Hénaff 1976) and 100,000 individuals in 1973 (Pichette and Beauchemin 1973). Both herds grew to large sizes up until the beginning of the 1990s when they peaked at more than one million individuals combined. They then experienced a continued decline and were last estimated at 199,000 individuals [90% CI = 183,080–214,920] in 2016 for the RAF (COSEWIC 2017), and 7200 individuals [90% CI = 6735–7665] in 2022 for the RG (Quebec government, unpublished data).

To assess the relationship between morphological traits and population size, we interpolated population estimates across the entire period when morphological data were available for each population. We first calculated a mean observation error (error calculation: CI/estimate × 100) based on surveys that had a confidence interval. We then applied this mean error to survey estimates that did not have a confidence interval (the mean errors were: Porcupine = 0.10, Beverly = 0.32, RAF = 0.46, RG = 0.33). We used estimates from a recent integrated population model (IPM) for the periods following 1991 and 1994 for the RG and RAF herds, respectively (Vuillaume 2023), to supplement our analyses. We used all population size data (field surveys + IPM) to generate a likely range of annual population estimates for each herd by fitting three locally‐weighted polynomial regressions using the *loess* function in R version 4.1.3 (R Core Team 2022): one across population estimates, one across the lower confidence interval bounds (LCI), and one across the upper confidence interval bounds (UCI; Figure 2). We adjusted the span of the *loess* to obtain the best fit of the smoothing curve to the estimates and CI bounds (estimated visually; Porcupine = 0.4; Beverly, RAF, and RG = 0.75). In years when both a field estimate and an IPM estimate were available, we used the lowest of the two LCIs and the highest of the two UCIs as the limits to the range of likely population sizes (Table A1.2).

### 2.3 Morphological Traits—Raw and Transformed Data

Morphological data included measurements of hind foot length, body mass, and body fat of individuals from the four studied herds over 33 years (1978–2010). Morphological data originated from various studies with different objectives, including assessments of physical characteristics (Couturier et al. 2009; Parker 1981), body composition (Huot 1989; Chan‐McLeod, White, and Russell 1999), habitat quality (Crête and Huot 1993), and maternal allocation to reproduction (Russell et al. 1998; Taillon et al. 2012). For the purposes of this study, we only considered individuals of known age and sex with at least one measurement of the three morphological traits.

Age was determined in the field using a tooth wear index (Hewison et al. 1999) or in the lab by measuring growth annuli on tooth sections. We used age to assign each caribou to a birth cohort. We separated individuals into three age classes: newborns (≤ 1 m.o.), yearlings (13–24 m.o.), and adults (≥ 25 m.o.). For the analysis of hind foot length, we considered caribou aged 2.5 years and older as adults because most individuals stop growing at that age (Parker 1981; Couturier et al. 2010). We carefully inspected the data set, removing aberrant values, duplicate entries and incomplete data, and four individuals that switched between the RAF and RG herds. We also removed adult and yearling males, which comprised < 6% of the total data set. We ignored them instead of combining them with females due to strong sexual dimorphism. This provided a sample of 4473 individuals measured for at least one trait (Table 1).

**TABLE 1:** Number of individuals measured for hind foot length (HFL), body mass, or body fat, and number of individuals that were measured for only one, two, or all three of these traits (and corresponding percentage) in the Porcupine, Beverly, Rivière‐aux‐Feuilles and Rivière‐George migratory caribou herds, 1978–2010.

Herd	HFL	Mass	Body fat	1 trait	2 traits	3 traits
Porcupine (*n = 283 ind*.)	225	282	227	28 (10%)	59 (21%)	196 (69%)
Beverly (*n = 708 ind*.)	656	698	603	37 (5%)	93 (13%)	578 (82%)
Rivière‐aux‐Feuilles (*n = 1056 ind*.)	987	794	95	331 (31%)	630 (60%)	95 (9%)
Rivière‐George (*n = 2426 ind*.)	1936	2009	353	860 (35%)	1260 (52%)	306 (13%)
Total (*n = 4473 ind*.)	3804	3783	1278	1256 (28%)	2042 (46%)	1175 (26%)

Across herds, morphological measurements were collected for different age and sex classes and in different seasons. In the Porcupine herd, data were collected on 283 adult females from 1987 to 1998, in March—June, September, and November. In the Beverly herd, 708 adult females were measured between 1980 and 1987, from 16 to 24 March and from 26 November to 13 December. Sampling of RAF caribou took place in 1988, 1991, and from 1994 to 2010. In this herd, 1056 individuals of different age and sex were measured in January—March, June—August, and October—November. In the RG herd, 2483 individuals of different age and sex were measured across all seasons in 1978–1981, 1983–1988, and 1992–2010. We excluded years when fewer than five individuals were measured (Zannèse et al. 2006). We also excluded trait‐herd combinations with less than 5 years of data.

Individuals were measured either alive or dead following sport hunting or scientific culls. Hind foot lengths (HFL) included direct measurements from the extremity of the calcaneus to the tip of the hoof [see Section 4 for possible discrepancies in methods among herds] (*n* = 2484) or were estimated from metatarsus length (*n* = 1320). We used a mixed linear regression to assess the relationship between the two measurements, based on 681 individuals that were measured for both hind foot and metatarsus lengths. We built various candidate models that considered the potential effect of sex and age, individually or in combination. We selected the most parsimonious model based on Akaike's information criterion corrected for small samples (AIC_c_; Burnham and Anderson 2002) using package *AICcmodavg* in R (Mazerolle 2023). We checked the normality of residuals and the linearity and homogeneity of variance in all models. The retained model was: ${\mathrm{HFL}}_{i}=-3.17+1.50*{\text{META}}_{i}+Ɛ.{\text{year}}_{i}$ where $Ɛ.{\text{year}}_{i}$ was the random effect of the year when the measurement was taken. We used this model to convert metatarsus lengths (${\text{META}}_{i}$) into hind foot length indices (${\mathrm{HFL}}_{i}$) for all individuals *i* that only had a metatarsus length.

The mass data were either from whole (*n* = 3756) or eviscerated individuals (*n* = 27, from the RG herd only). Similar to the procedure for HFL data, we used a mixed linear regression to assess the relationship between live and eviscerated mass based on 397 individuals that were weighed before and after evisceration. The model selection process was the same as for HFL, but here we retained the second‐best model because it was within $\Delta_{\text{AICc}}$ < 2 and contained fewer parameters compared to the top model (see Arnold 2010). We transformed eviscerated mass (${\text{MASS}}_{\text{evisc},i}$) into live mass (${\text{MASS}}_{\text{live},i}$) using the retained model which included age class as a fixed effect and year as a random effect: ${\text{MASS}}_{\text{live},i}=13.33+{\mathrm{Age}}_{i}+1.27*{\text{MASS}}_{\text{evisc},i}+Ɛ.{\text{year}}_{i}$ where ${\mathrm{Age}}_{i}$ was the effect of age class.

The body fat indices for the Beverly, RAF, and RG herds (*n* = 1051) were based on a kidney‐femur fat index combining perinephric fat mass from both kidneys, average kidney mass, and femur marrow fat percentage as described in Couturier et al. (2009). For Porcupine caribou, the body fat index was computed from percent total body water estimated from tissue samples using equations in Allaye‐Chan (1991; *n* = 227).

**FIGURE 3:**
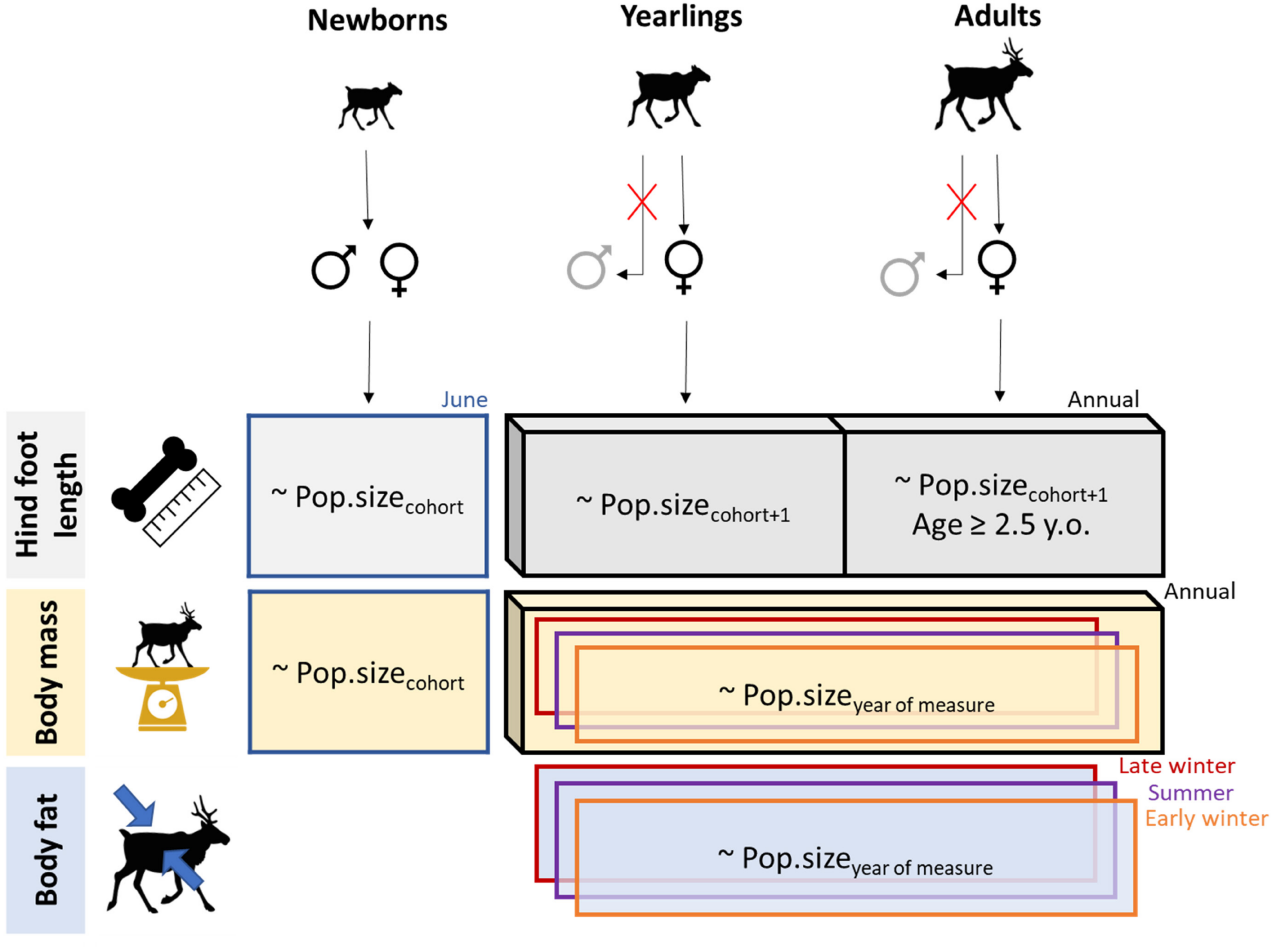
Synthesis diagram of the data used in this study. Hind foot length (cm), body mass (kg) and body fat index were measured in 4 migratory caribou (*Rangifer tarandus*) herds across Canada and Alaska, for different age‐sex classes. Relationships were tested with population size at the year of cohort, cohort+1 or year of measurement, depending on the trait. Note that for newborns, year of measurement and cohort were confounded, and that only June measurements were considered. Three seasonal collection periods were defined for body mass and body fat of yearlings and adults: Late winter (January–April), summer (May–August), and early winter (September–December).

We evaluated the relationship between morphological traits and population size annually and/or seasonally, depending on the trait. We distinguished three seasonal collection periods for adults and yearlings: summer (May–August), early winter (September–December), and late winter (January–April). For newborns, we restricted all analyses to the month of birth (June; Figure 3). Being a skeletal measurement, the HFL reflects conditions experienced during an individual's growth phase; we thus evaluated the relationship between HFL and population size in cohort year +1, except for newborns that were only assessed in their month of birth. Fat reserves in ungulates largely depend on seasonal forage and weather conditions (Parker, Barboza, and Gillingham 2009), we thus restricted the evaluation of a potential link between population size and body fat at the seasonal scale. We assessed relationships with body fat values during all seasonal periods, including early and late winter, because contrary to most northern mammals that usually only gain fat during summer, migratory caribou may also gain fat during winter (Couturier et al. 2009). Finally, we assessed the relationship between mass and population size both annually and seasonally. For all seasonal analyses, we only considered season × herd combinations with at least 30 measured individuals.

Body mass and body fat can vary extensively during a given year, such that traits measured in summer are not directly comparable to traits measured in winter. Thus, to assess the relationship between morphological traits and population size across years, we required comparable intra‐annual and intra‐seasonal morphometric measurements. To do so, we adjusted trait values to the mean date of measurement at the annual or seasonal scales using linear models to determine the best correction to apply (Table A2.2). For each herd × trait combination, we modeled the trait as a function of the Julian day when the measurement was taken. We tested the effect of date using all increments from 1‐ to 5‐order polynomial and selected the model providing the best fit using an ANOVA (Pr(> F)), checking all model assumptions (additional details are provided in Appendix 2: Table A2.1).

### 2.4 Analytical Framework

To evaluate the relationship between population size and the three morphological traits across herds, age‐sex classes, and seasons, we used linear mixed regressions integrated in a Bayesian modeling framework. We fitted separate models for each herd. We used the three morphological traits as response variables and population size as explanatory variable in all models, as we aimed to assess whether morphological traits reflected changes in population size. Each morphological trait (${\text{Trait}}_{i}$) was expressed as a combination of the mean value of the trait ($\mu_{\text{year}}$) for the corresponding herd × year combination and a linear predictor of annual random interindividual variability to account for different individuals being sampled each year and the fact that some individuals were sampled over several years (${Ɛ}_{i,\text{year}}$, Equation 1).

(1) $${\text{Trait}}_{i}=\mu_{\text{year}}+{Ɛ}_{i,\text{year}}$$

We expressed $\mu_{\text{year}}$ as a combination of an intercept (α) equal to the mean value of the trait over the study period and a fixed effect (β) of population size during the year of the cohort+1 or year of measurement ($\mathrm{Pop}.{\text{size}}_{\text{year}}$), depending on the trait (Equation 2).

(2) $$\mu_{\text{year}}=\alpha +\beta *\mathrm{Pop}.{\text{size}}_{\text{year}}$$

When morphological data were available for both growth and decline phases of the population, with at least 5 years in each phase, we included population phase in models (Bonenfant et al. 2009) in interaction with population size by adjusting the fixed effect of population size for population phase ($\beta_{\text{phase}}$; Equation 3).

(3) $$\mu_{\text{year}}=\alpha +\beta_{\text{phase}}*\mathrm{Pop}.{\text{size}}_{\text{year}}$$

To test for potential seasonal differences in the relationship between the population size and individual body mass or body fat, we allowed the intercept ($\alpha_{\text{season}}$) and the fixed effect of population size ($\beta_{\text{season}}$) to vary across all seasons for which we had sufficient data (Equation 4).

(4) $$\mu_{\text{year}}=\alpha_{\text{season}}+\beta_{\text{season}}*\mathrm{Pop}.{\text{size}}_{\text{year}}$$

We assessed annual and seasonal variations in separate models. Population size ($\mathrm{Pop}.{\text{size}}_{\text{year}}$) followed a Normal distribution centered on the observed mean population estimates from the locally weighted polynomial regression (*loess*) fitted across the observed mean of population estimates determined from field estimates (and the IPM for RAF and RG; Equation 5).

(5) $$\mathrm{Pop}.{\text{size}}_{\text{year}}\sim \text{Normal}\;\left(\mu_{\mathrm{pop}.\mathrm{est},\text{year}},\sigma_{\mathrm{pop}.\mathrm{est},\text{year}}\right)$$

We estimated the standard deviation of this distribution based on the 95% CI of the annual population estimates, allowing the population size to take a value in this interval at each iteration.

We used uninformative priors for the fixed effect of population size (β), allowing for either a negative or positive relationship with a *Uniform* distribution between −1 and 1, and for the random effect of individual‐year (Ɛ). We used weakly‐informative priors for the intercepts (α) by choosing a uniform distribution bounded between realistic values according to each morphological trait (Table A3.1). We considered estimates whose CI did not overlap zero as statistically significant relationships between a response variable (here, one of the three morphological traits) and population size. We fitted Bayesian models using the software Jags v.4.2.0 (Plummer 2015), called in R using package *R2jags* (Yu‐Sung and Yajima 2015). We ran 3 Markov chain Monte Carlo (MCMC) chains of 100,000 iterations and discarded the first 60,000 iterations as burn‐ins with a thinning of 10. We confirmed the convergence of MCMC chains using the Brooks Gelman–Rubin convergence statistic, Rhat< 1.1 which represents the potential scale reduction factor (Brooks and Gelman 1998).

## 3 Results

All annual results for HFL, body mass, and seasonal results for body fat are in Table 2. Seasonal results for body mass are in Table A4.3; they seldom differed from the annual results, therefore we do not repeat them here. We observed high variability across herds, age classes, phases and seasons. Intra‐annual variability of traits was lower than inter‐annual variability among the four herds. The herds with the heaviest individuals did not necessarily have the largest or fattest individuals. General descriptive statistics on morphometrics of the three morphological traits, for each age class and each herd are reported in Table A4.1,2.

**TABLE 2:** Relationships between estimated population size and morphological traits in 4 migratory caribou herds across Canada and Alaska, according to age class, sex and season. Morphological traits include hind foot length (cm), body mass (kg) and a body fat index. We distinguished three data collection seasons: Summer (May–August), early winter (September–December), and late winter (January–April). Rhat is the potential scale reduction factor and is an indicator of successful convergence. n.eff is an estimate of the number of independent samples from the posterior distribution. We highlighted in bold the significant effects of population size on morphological traits, i.e. those whose condience interval does not overlap 0.

Herd	Age class	Morphological trait	No. individuals	No. years	Parameter	Parameter estimate	LCI	UCI	Rhat	n.eff
Porcupine	Adult	Hind foot length	189	13	α	45.70	44.77	46.09	1.00	7400
					β	**−0.32**	**−0.35**	**−0.26**	1.00	4900
					σ	1.80	1.63	2.00	1.00	12,000
		Mass	278	12	α	82.98	78.23	91.95	1.00	1500
					β	**0.72**	**0.17**	**0.99**	1.02	1900
					σ	9.94	9.12	10.83	1.00	12,000
		Fat (*Early winter*)	125	8	α	10.67	7.88	13.56	1.02	420
					β	0.00	−0.18	0.17	1.02	370
					σ	1.56	1.41	1.73	1.00	5300
		Fat (*Late winter*)	71	6	α	14.50	11.37	16.45	1.00	12,000
					β	−0.12	−0.24	0.07	1.00	12,000
					σ	1.56	1.41	1.73	1.00	5300
Beverly	Adult	Hind foot length (*during population decline*)	322	9	α	36.61	36.10	37.04	1.00	2700
					β	0.02	−0.01	0.05	1.00	3500
					σ	1.32	1.25	1.39	1.00	12,000
		Hind foot length (*during population increase*)	327	6	α	36.61	36.10	37.04	1.00	2700
					β	**0.04**	**0.02**	**0.07**	1.00	2900
					σ	1.32	1.25	1.39	1.00	12,000
		Mass	698	8	α	69.65	63.70	74.03	1.00	1800
					β	**0.61**	**0.40**	**0.92**	1.00	1700
					σ	7.90	7.49	8.34	1.00	12,000
		Fat (*Early winter*)	161	5	α	11.28	9.00	13.56	1.00	710
					β	0.00	−0.11	0.10	1.00	730
					σ	2.31	2.19	2.45	1.00	9200
		Fat (*Late winter*)	442	8	α	9.78	8.04	11.06	1.00	1400
					β	**0.15**	**0.09**	**0.24**	1.00	1800
					σ	2.31	2.19	2.45	1.00	9200
Rivière‐aux‐Feuilles	Adult	Hind foot length	197	17	α	55.26	54.22	56.29	1.00	1100
					β	0.00	−0.01	0.02	1.00	1200
					σ	1.55	1.40	1.71	1.00	12,000
		Mass	163	5	α	83.09	77.88	88.51	1.00	3200
					β	−0.09	−0.21	0.02	1.00	4400
					σ	7.87	7.07	8.80	1.00	12,000
	Yearling	Hind foot length	99	9	α	47. 62	46.13	49.16	1.00	12,000
					β	0.02	−0.02	0.05	1.00	12,000
					σ	1.76	1.53	2.04	1.00	12,000
		Mass	87	9	α	38.85	34.14	43.39	1.00	11,000
					β	0.02	−0.08	0.12	1.00	12,000
					σ	5.01	4.32	5.85	1.00	12,000
	Newborn	Hind foot length (*June*)	236	13	α	34.76	33.83	35.72	1.00	5700
					β	**−0.03**	**−0.05**	**−0.02**	1.00	6400
					σ	1.89	1.72	2.07	1.00	12,000
		Mass (*June*)	276	14	α	5.49	5.06	5.90	1.00	2100
					β	0.00	−0.01	0.01	1.00	1800
					σ	0.97	0.89	1.05	1.00	9600
Rivière‐George	Adult	Hind foot length (*during population increase*)	256	13	α	55.71	55.17	56.24	1.00	2100
					β	0.00	−0.01	0.01	1.00	2100
					σ	1.91	1.79	2.03	1.00	6200
		Hind foot length (*during population decline*)	197	16	α	55.71	55.17	56.24	1.00	2100
					β	0.00	−0.01	0.01	1.00	2200
					σ	1.91	1.79	2.03	1.00	6200
		Mass (*during population increase*)	258	5	α	93.18	90.08	96.26	1.00	6800
					β	**−0.12**	**−0.18**	**−0.06**	1.00	8500
					σ	10.18	9.51	10.92	1.00	12,000
	
		Mass (*during population decline*)	160	5	α	93.18	90.08	96.26	1.00	6800
					β	**−0.21**	**−0.32**	**−0.09**	1.00	9200
					σ	10.18	9.51	10.92	1.00	12,000
		Fat (*Early winter*)	110	7	α	6.76	4.96	8.67	1.00	4100
					β	0.00	−0.03	0.03	1.00	6900
	σ				2.28	2.08	2.50	1.00	12,000
	Yearling	Hind foot length	196	18	α	49.33	48.67	49.98	1.00	11,000
					β	0.01	−0.01	0.02	1.00	12,000
					σ	2.00	1.81	2.22	1.00	4400
		Mass	172	17	α	45.43	43.63	47.23	1.00	12,000
					β	**−0.06**	**−0.10**	**−0.02**	1.00	6000
					σ	5.31	4.77	5.92	1.00	4800
	Newborn	Hind foot length (*June*)	459	15	α	32.85	32.08	33.59	1.00	3500
					β	0.00	−0.01	0.01	1.00	3800
					σ	1.98	1.85	2.11	1.00	7900
		Mass (*June, during population increase*)	209	6	α	5.94	5.73	6.14	1.00	4800
					β	**0.01**	**0.01**	**0.02**	1.00	4400
					σ	1.18	1.13	1.23	1.00	12,000
		Mass (*June, during population decline*)	711	17	α	5.94	5.73	6.15	1.00	4800
					β	0.00	−0.01	0.00	1.00	5000
σ					1.18	1.13	1.23	1.00	12,000

Abbreviations: α = intercept of the regression, β = effect of population size on the morphological trait, σ = random effect of individuals, LCI = lower bound of 95% confidence interval, UCI = upper bound of 95% confidence interval.

### 3.1 Porcupine Herd

**FIGURE 4:**
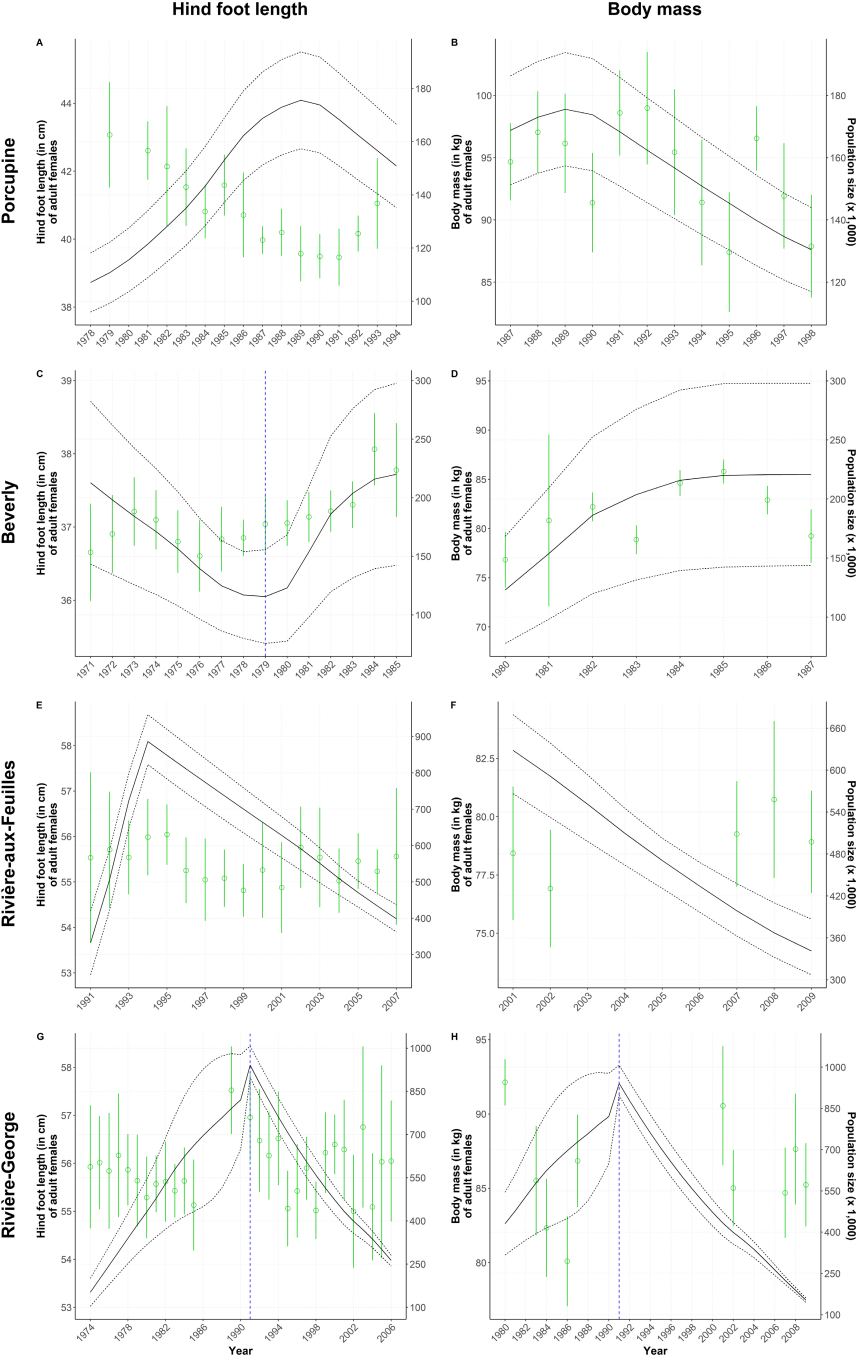
Annual average values (with standard deviation) of morphological traits measured on adult female caribou (*Rangifer tarandus*) from the Porcupine, Beverly, Rivière‐aux‐Feuilles, and Rivière‐George migratory herds. Hind foot length (cm) and body mass (kg) are overlaid with population estimates (solid lines) and their 95% confidence intervals (dotted lines). A change in population phase (i.e., growth vs. decline) using a blue dashed line is indicated only when it was retained in the most parsimonious model. (A) Hind foot length and (B) body mass of adult females of the Porcupine herd; (C) hind foot length and (D) body mass of adult females of the Beverly herd; (E) hind foot length and (F) body mass of adult females of the Rivière‐aux‐Feuilles herd; (G) hind foot length and (H) body mass of adult females of the Rivière‐George herd.

The hind foot length of adult females from the Porcupine herd decreased with increasing population size (−0.32 [−0.35; −0.26]; Figure 4A). An increase in population size by 50,000 individuals led to an average hind foot length decrease of 3 cm (ΔY = ‐3 cm for Δ*X* = 50,000 individuals). On the contrary, body mass increased with population size (0.72 [0.17; 0.99], Δ*Y* = 4 kg for Δ*X* = 50,000 individuals; Figure 4B). This relationship between body mass and population size at the annual level was driven by data collected in early winter but not those collected in late winter, as determined using seasonal analyses. We found no relationship between the body fat of adult females and population size in this herd.

### 3.2 Beverly Herd

During the population growth phase (1980–1985), the hind foot length of adult females from the Beverly herd increased with population size (0.04 [0.02; 0.07], Δ*Y* = 0.5 cm for Δ*X* = 120,000 individuals; Figure 4C). This relationship was not observed during the preceding decline phase (1971–1979). The body mass of adult females also increased with population size (0.61 [0.40; 0.92], Δ*Y* = 3 kg for Δ*X* = 50,000 individuals), a relationship that was confirmed at the annual scale (Figure 4D) and for both early (0.31 [0.04; 0.65]) and late winter (0.69 [0.46; 0.97]). The body fat of adult females increased with population size during late winter (0.15 [0.09; 0.24], Δ*Y* = 1 for Δ*X* = 60,000 individuals) but not early winter.

### 3.3 Rivière‐Aux‐Feuilles (RAF) Herd

**FIGURE 5:**
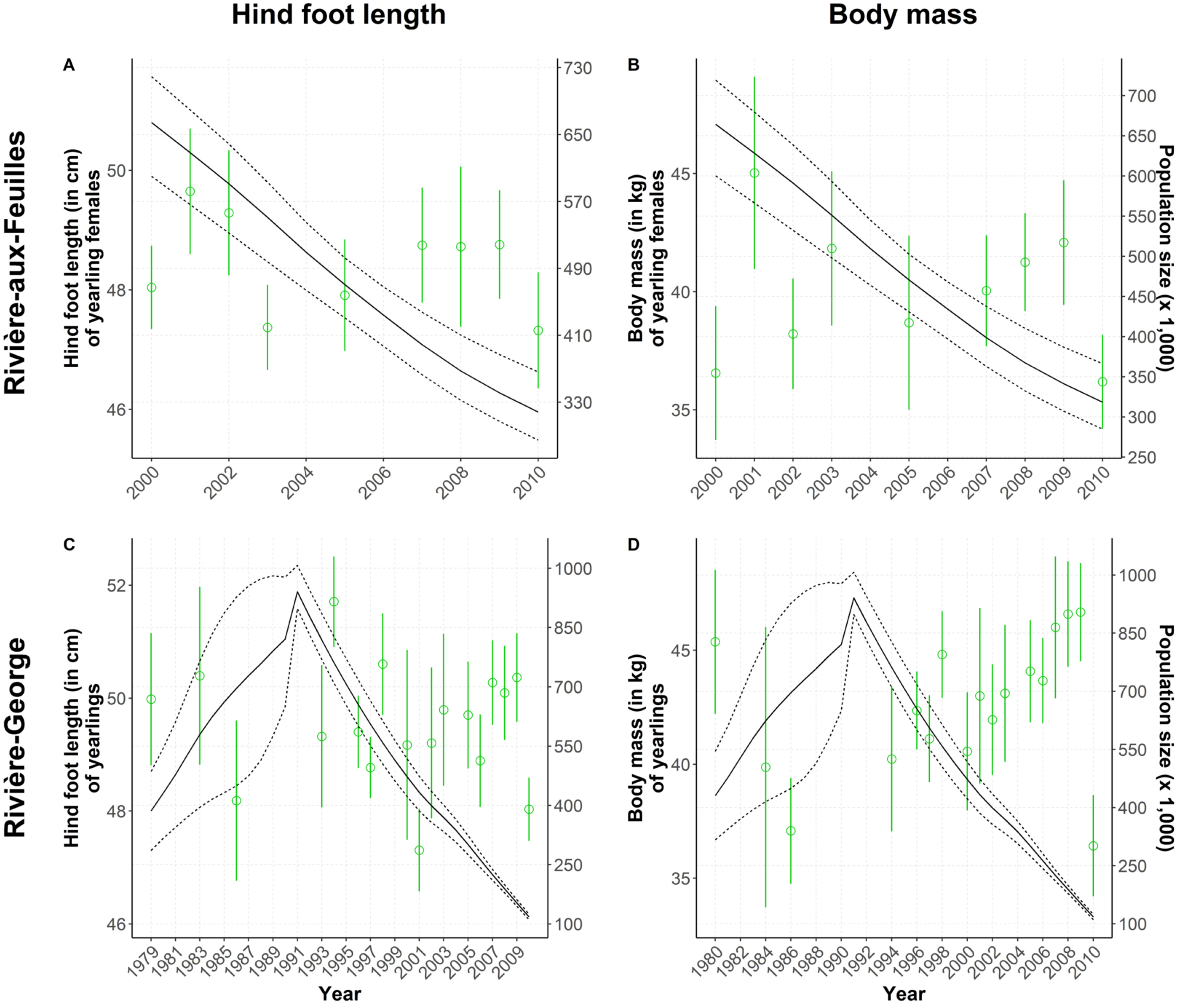
Annual average values (with standard deviation) of morphological traits measured on yearling females from the Rivière‐aux‐Feuilles and Rivière‐George migratory caribou (*Rangifer tarandus*) herds. Hind foot length (cm) and body mass (kg) are overlaid with population estimates (solid lines) and their 95% confidence intervals (dotted lines). (A) Hind foot length and (B) body mass of yearlings of the Rivière‐aux‐Feuilles herd; (C) hind foot length and (D) body mass of yearlings of the Rivière‐George herd.

**FIGURE 6:**
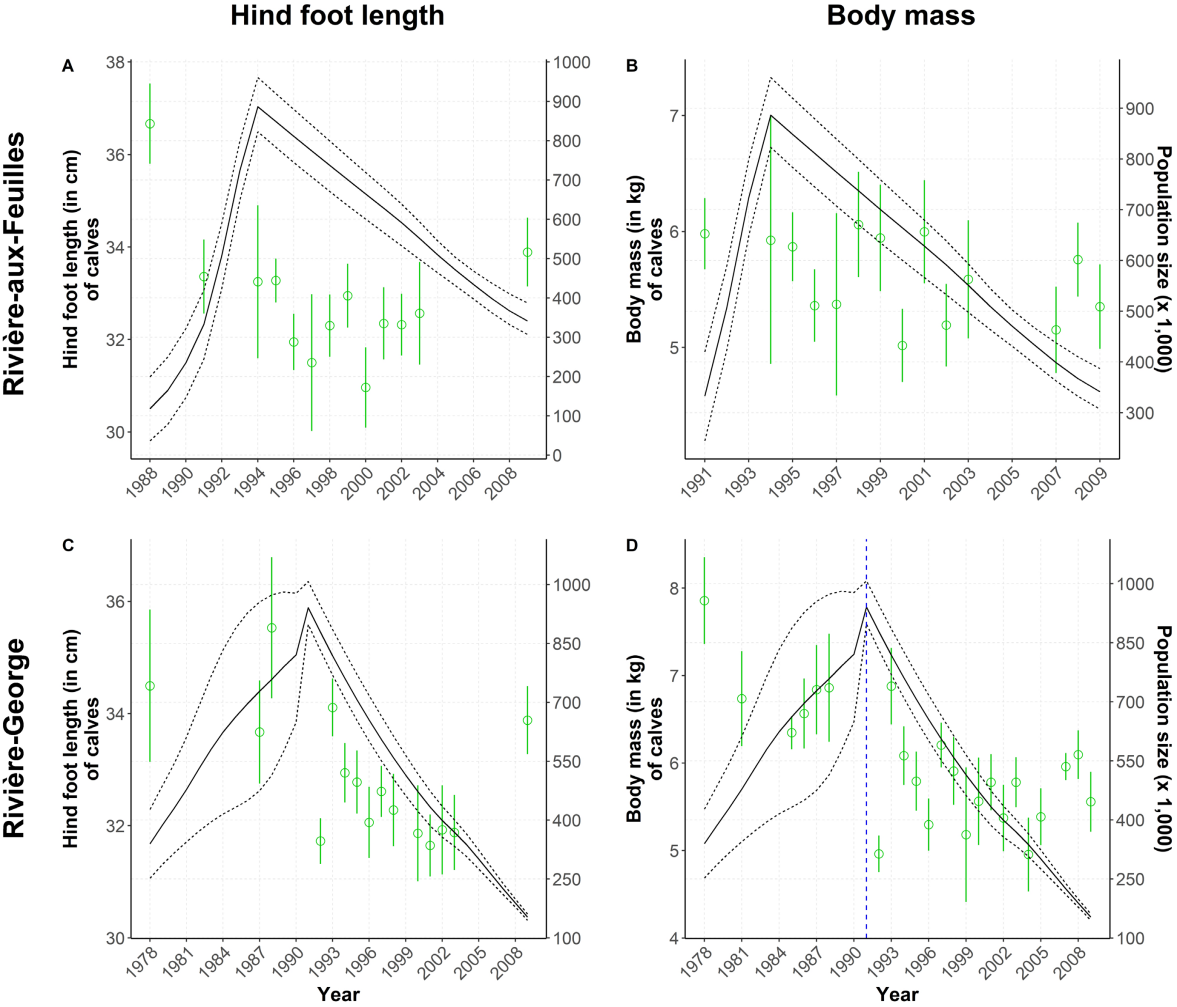
Annual average values (with standard deviation) of morphological traits measured on newborn males and females from the Rivière‐aux‐Feuilles and Rivière‐George migratory caribou (*Rangifer tarandus*) herds. Hind foot length (cm) and body mass (kg) are overlaid with population estimates (solid lines) and their 95% confidence intervals (dotted lines). We indicate a change in population phase (i.e., growth vs. decline) using a blue dashed line only when it was retained in the most parsimonious model. (A) Hind foot length and (B) body mass of newborns of the Rivière‐aux‐Feuilles herd; (C) hind foot length and (D) body mass of newborns of the Rivière‐George herd.

We found no relationship between the morphological traits of either yearlings or adults from the RAF herd and population size, regardless of the temporal scale assessed (respectively Figures 5A,B and 4E,F). The hind foot length of newborns was negatively related to population size (−0.03 [−0.05; −0.02], Δ*Y* = −0.9 cm for Δ*X* = 300,000 individuals; Figure 6A). There was no relationship between the body mass of newborns and population size (Figure 6B).

### 3.4 Rivière George (RG) Herd

The body mass of all age classes was related to population size in this herd, but the direction of the relationships varied (Adult females: −0.12 [−0.18; −0.06] in growth phase and − 0.21 [−0.32; −0.09] in decline phase, Figure 4H; yearlings: −0.06 [−0.10; −0.02], Figure 5D; calves: 0.01 [0.01; 0.02], Figure 6D). The body mass of yearlings and adult females was negatively related to population size, whereas the inverse occurred for calves, but only during the declining phase in 1991–2010. For adult females, this means that an increase in population size of 200,000 individuals during the growth phase could lead to an average mass decrease of 3 kg, while a similar decline during the decline phase could be reflected on an average mass increase of 4 kg. This negative relationship observed during both growth and decline phases was confirmed for the early (−0.10 [−0.19; −0.02]) and late winter seasons (−0.30 [−0.49; −0.14]), but not during summer. The hind foot length and body fat of individuals from all age classes were not linked to population size in this herd (adults, Figure 4G; yearlings, Figure 5C; calves, Figure 6C).

## 4 Discussion

Wildlife managers require precise estimates of abundance to determine levels of harvest or establish protection measures to maintain healthy populations of wild large herbivores. Unfortunately, they sometimes need to make these decisions in the absence of reliable demographic data. In years when population surveys are not available, managers need alternative ecological indicators, easier to collect, that can inform on population trends. Using a Bayesian modeling framework, we evaluated whether hind foot length, body mass, and body fat, three morphological traits often measured by wildlife managers, were related to population size in four migratory caribou herds distributed across northern North America over 33 years. The analyses suggested that morphological traits were not consistently linked to population size. What relationships existed varied according to trait, age class, and herd. In some herds (e.g., Beverly), we found relatively strong relationships between morphological traits and population size for some age classes, but several of those relationships were opposite to the expectation of a density‐dependent negative effect on morphology. In other herds, we found only weak or no relationship.

Previous studies observed a negative relationship between hind foot length and population size in white‐tailed deer (Ashley, McCullough, and Robinson 1998) and caribou (Klein 1968). Zannèse et al. (2006) also found that the hind foot length of roe deer in southwestern France declined with increasing population size. We expected that the hind foot length of individuals would be a good predictor of population size during early development, but this trait was related to population size in only 3 of the 8 age class × herd combinations we assessed. Significant relationships were only observed during population growth phases, but were not always negative. Hind foot lengths were smaller for individuals born in years of high population size in the Porcupine and Rivière‐aux‐Feuilles herds (for calves only), but the opposite was true for the Beverly herd.

Similarly to hind foot length, body mass was previously shown to be a good predictor of population size in many large herbivores, including caribou (Toïgo et al. 2006; Couturier et al. 2009; Taillon et al. 2011). Other Arctic herbivores, however, showed no relationships between body condition and population growth (*Branta leucopsis*, Layton‐Matthews et al. 2021). We found no systematic relationship between body mass and population size, as the direction and strength of the few statistically significant relationships varied across herds, age classes, and population phases.

Like HFL and body mass, body fat was also shown to be a good index of body condition in caribou in previous studies (Couturier et al. 2009; Parker, Barboza, and Gillingham 2009; Taillon et al. 2011). Yet, here, the only relationship we identified with population size was a positive relationship in late winter for adult females from the Beverly herd. Sparse and limited data on body fat, with sometimes 10–20 years between measurements (e.g., Porcupine 1987 vs. 1997, RG 1983 vs. 2002), might have reduced our capacity to detect relationships between this morphological trait and population size.

Our Bayesian modeling framework should circumvent some of the issues that typically influence attempts to link individual morphology to demography. For example, we used a wide range of likely values for population size, bounded by the limits of the confidence interval, rather than a single value, thereby accounting for the large uncertainty often associated with population estimates. The smoothed *loess* curve for population size was a realistic representation of changes in abundance for a long‐lived species like caribou which may display prolonged time lags in demographic responses to the environment (Russell and White 2000). Finally, by integrating the three linear mixed regressions linking the three morphological traits to population size in the same model, we were better equipped to compare the relative strength of the relationships between the three assessed traits and population size. Yet, we found few statistically significant relationships.

### 4.1 The Influence of the Demographic Phase

Few of our results supported our initial predictions that hind foot length, body mass, and body fat of individuals should decrease with increasing population size. These predictions were based on the assumption that population size was correlated to the strength of intraspecific competition. The effects of population phase may provide a potential explanation as to why so few morphological traits were linked to population size. Populations typically experience four demographic phases: growing at low density, growing at high density, declining at high density, and declining at low density. For the same population size, environmental conditions and their impacts on individuals can be different depending on the demographic phase.

As a first example, we focus on the Beverly herd, where, contrary to our initial prediction, we found a positive relationship between the body mass of adult females and population size. In this herd, measurements were taken mainly during a population growth phase at relatively low population size. During this phase, body mass could increase with population size as long as the access to high‐quality resources is not limited by intraspecific competition and females are in good body condition (Gaidet and Gaillard 2008), have high fecundity (Borowik and Jędrzejewska 2018), and give birth to large offspring (Taillon et al. 2012). If individuals at the beginning of the growing phase were small as a residual effect of the preceding decline phase, then this positive relationship would be expected to persist until intraspecific competition becomes strong enough to reduce individual food intake. Only then should we expect to observe a negative effect of population size on body condition.

In the Rivière‐aux‐Feuilles herd we found no relationship between morphological traits in adults and yearlings and population size. In this herd, most measurements were taken during a decline phase at low population size. Relationships between body mass and population size during that phase are expected to be weaker because a progressively smaller number of individuals are competing for resources depleted by high densities in the preceding years. During that phase, the body condition of animals could be mostly determined by density‐independent factors such as severe weather (Bowyer et al. 2014). The absence of a relationship for body mass of calves of the RAF herd, despite many measurements taken during the high‐density declining phase, confirms the findings by Taillon et al. (2012) in the same population, that maternal mass may mask the effects of population size on calf body mass. These observations indicate that other variables such as access to food or maternal condition could modulate the relationship between morphological traits and population size during certain demographic phases.

Unfortunately, comparable datasets about long‐term population trends and other environmental factors are often lacking and therefore cannot inform the management of large herbivores in northern ecosystems. We included information about demographic phase in our models when possible, although we limited these phases to growth vs. decline due to limited sampling. We recognize that including such information might prove difficult for the prospective management of large herbivore populations, especially in areas where demographic trends are unknown. We had 30 years of data for the Rivière‐George herd, yet that was insufficient to clearly link morphological traits to abundance across demographic phases. Lack of data will continue to hinder statistical testing of the relationship between body condition and abundance for long‐lived species like caribou, underlining the importance of continued long‐term monitoring (Lindenmayer, Lavery, and Scheele 2022). Considering our results and the difficulties exposed above, we conclude that, in the absence of empirical data on population trends, morphological traits cannot predict population size.

### 4.2 Field Constraints and Other Considerations

A strength of our study was its very large spatiotemporal scale, with more than 30 years of data collected across four distinct populations. There were, however, problems of inconsistent data collection and possible observer bias, which cannot be easily quantified. Our data suggest that the hind foot length of adult females from the Beverly herd were 33.5% shorter on average than for adult females from the Rivière‐George herd, a biologically unlikely difference of about 19 cm. These differences should have resulted in major differences in body mass, which we did not observe, suggesting that the hind feet were measured differently by various investigators. Garel et al. (2010) recommended using standardized tools to increase consistency in leg measurements; such tools were not used during data collection in this study. The attributes of some morphological traits may also have been biased by measurement errors. Martin et al. (2013) concluded that measurement errors could reach 2.7 cm, or 66% of among‐individual variation in hind foot length measurements in long‐term studies of mountain goats (*Oreamnos americanus*) and bighorn sheep (*Ovis canadensis*). Because of these factors, we refrained from comparing morphological traits among herds.

Other aspects of our study may have confounded the relationship between individual morphometrics and population dynamics. Using individual data to infer biological response at the population level is a challenge, particularly for herds that have very large size and are distributed over a massive range. The sample sizes of morphometric data in some years may not have been sufficient to reflect existing individual heterogeneity.

We assumed that population size was related to density, but population growth may not always lead to an increase in density (Gaston et al. 2000). Caribou are a highly mobile species, and individuals may limit intraspecific competition by moving into new areas to access food at high population abundance. Thus, increases in population size may not always have been linked to decreases in individual access to resources. Finally, environmental conditions were likely highly variable among and within herds across the broad spatial (> 4000 km) and temporal (> 30 year) scales used in this study. Determining the extent to which external factors influenced our results would require additional data.

### 4.3 Recommendations for Future Research

Reliability of morphological data should be the first concern for future research. One approach could involve optimizing the representation of morphometric heterogeneity within herds by improving data collection, accounting for all possible sources of bias including rigorous methodology and avoiding selective sampling. New approaches could improve morphometric datasets. For example, trail cameras would be less selective than hunting on individual sampling. Images can now be used to accurately estimate basic morphometry (length, height) of individuals (Leorna, Brinkman, and Fullman 2022). These methods could considerably increase sample size on certain traits without the need to rely on handling of individuals. Traits such as fat content, however, cannot be assessed from images.

When analyzing the response of morphometric traits, researchers should consider other sources of variability unrelated to changes in population size or environmental conditions, such as selective harvests (by hunters or scientists) or herd mixing (Prichard et al. 2020), which can mask density‐dependent responses. Individual heterogeneity can also vary widely between herds depending on the historical context. For example, introduced populations may show major morphometric variability that could persist over the long term (Mager, Colson, and Hundertmark 2013).

Researchers seeking to recover populations that have declined, or predict population changes in *Rangifer* should bear in mind that even the best morphometric data do not adequately predict population size (Traill et al. 2021). If population surveys are too few and widely spaced over time, morphological traits will not inform on population size. Morphological traits are still useful proxies of other factors explaining the fecundity and survival of large herbivores (Festa‐Bianchet, Jorgenson, and Réale 2000; Gaillard et al. 2000), which contribute to population growth. For migratory caribou, strong empirical information would be particularly critical, as many populations are in decline (Vors and Boyce 2009).

## Author Contributions

**Barbara Vuillaume:** data curation (equal), formal analysis (lead), investigation (equal), methodology (lead), visualization (equal), writing – original draft (equal), writing – review and editing (equal). **Mathieu Leblond:** conceptualization (equal), data curation (equal), formal analysis (supporting), funding acquisition (equal), investigation (equal), methodology (supporting), project administration (lead), resources (equal), supervision (lead), validation (equal), visualization (equal), writing – original draft (equal), writing – review and editing (equal). **Marco Festa‐Bianchet:** conceptualization (equal), funding acquisition (equal), investigation (equal), validation (equal), writing – review and editing (equal). **Steeve D. Côté:** conceptualization (equal), funding acquisition (equal), project administration (supporting), resources (equal), validation (equal), writing – review and editing (equal).

## Ethics Statement

All applicable international, national, and institutional guidelines for the care and use of animals were followed by the original investigators during data collection. This article does not contain any studies with human participants performed by any of the authors.

## Conflicts of Interest

The authors declare no conflicts of interest.

## Supporting information


Appendix S1.

Appendix S2.

Appendix S3.

Appendix S4.

## Data Availability

The demographic datasets analyzed in the current study are included in this published article and its appendices. Code for statistical analysis and the morphological datasets analyzed in the current study have been deposited in Dryad, http://datadryad.org/stash/share/5oXHIoOoy_d2Nau6tXX59JhZ‐PJRw4mhcpPyxGwUR4A, and will be published upon acceptance of the paper.
