# Supplementary material for: Morphological Traits Are Not Consistently Related to Population Size in Four Migratory Caribou Populations Across North America
Source: Ecol Evol. 2024 Oct 15;14(10):e70468. doi: 10.1002/ece3.70468 (PMC11480523; doi:10.1002/ece3.70468)
Supplement: Supplementary file 1 — Appendix S1. [file ECE3-14-e70468-s002.docx]

**APPENDIX 1:**

**Population size estimates**

**1. Population size estimates from surveys**

**Table A.1.1.** Estimates of population size with their confidence intervals (CI) for 4 migratory caribou (*Rangifer tarandus*) herds in Canada and Alaska between 1971 and 2010. The CI are 95% for the Porcupine and Beverly herds, and 90% for the Rivière-aux-Feuilles and Rivière-George herds.

| **Herd** | **Year** | **Population estimate** | **Confidence interval (90 or 95%)** | **Methodology** | **References** |
| --- | --- | --- | --- | --- | --- |
| Porcupine | 1972 | 102,000 | NA | Photographic^a^ | Urquhart, 1983 |
|  | 1977 | 105,000 | NA | Photographic | Fancy et al., 1994 |
|  | 1979 | 110,000 | NA | Photographic | Fancy et al., 1994 |
|  | 1983 | 135,000 | NA | Photographic | Fancy et al., 1994 |
|  | 1987 | 165,000 | NA | Photographic | Arthur et al., 2003; Fancy et al., 1994 |
|  | 1989 | 178,000 | NA | Photographic | Arthur et al., 2003; Fancy et al., 1994 |
|  | 1992 | 160,000 | NA | Photographic | Arthur et al., 2003; Fancy et al., 1994 |
|  | 1994 | 152,000 | NA | Photographic | Arthur et al., 2003 |
|  | 1998 | 129,000 | NA | Photographic | Stephenson 1999; Arthur et al., 2003 |
|  | 2001 | 123,000 | NA | Photographic | Caikoski, 2020 |
|  | 2010 | 169,000 | 15,507 | Photographic | Caikoski, 2020 |
|  | 2013 | 197,228 | 28,561 | Photographic | Porcupine Caribou Technical Committee, 2021 |
|  | 2017 | 218,000 | 15,894 | Photographic | Porcupine Caribou Technical Committee, 2021 |
|  |  |  |  |  |  |
| Beverly | 1971 | 210,000 | NA | Visual^b^ | Gunn & Decker, 1982 |
|  | 1974 | 180,000 | NA | Visual | *Government of the Northwest Territories* |
|  | 1978 | 130,000 | NA | Visual | Heard, 1982; Heard & Decker, 1980 |
|  | 1980 | 110,000 | NA | Visual | Gunn & Decker, 1982 |
|  | 1982 | 164,338 | 72,332 | Photographic | Heard & Jackson, 1990; Stephenson et al., 1984 |
|  | 1984 | 263,691 | 80,652 | Photographic | Heard & Jackson, 1990 |
|  | 1987 | 189,561 | 70,961 | Photographic | Heard & Jackson, 1990 |
|  | 1994 | 276,000 | 106,600 | Photographic | Williams, 1995 |
|  | 2011 | 124,200^c^ | 14,000^c^ | Photographic | Campbell et al., 2012 |
|  |  |  |  |  |  |
| Rivière-aux- | 1975 | 56,000 | NA | Visual | Le Hénaff, 1976 |
| Feuilles | 1983 | 101,000 | 43,430 | Visual | Le Hénaff, 1983 |
|  | 1986 | 121,000 | 56,000 | Photographic | Crête et al., 1987 |
|  | 1991 | 276,000 | 75,900 | Photographic | Couturier, 1994 |
|  | 2001 | 628,000^d^ | -^d^ | Photographic | Couturier et al., 2004 |
|  | 2011 | 430,000 | 98,900 | Photographic | Taillon et al., 2016 |
|  | 2016 | 199,000 | 15,920 | Photographic | COSEWIC, 2017 |
|  |  |  |  |  |  |
| Rivière- | 1973 | 100,000 | NA | Visual | Pichette & Beauchemin, 1973 |
| George | 1976 | 263,000 | NA | Visual | Luttich, 1976; Messier et al., 1988 |
|  | 1980 | 390,000 | 85,000 | Visual | Juniper, 1980 |
|  | 1984 | 643,600 | 161,000 | Photographic | Crête et al., 1991; Goudreault et al., 1985 |
|  | 1988 | 682,100^e^ | 246,000^e^ | Photographic | Crête et al., 1989, 1991 |
|  | 1993 | 823,000^e^ | 104,000^e^ | Photographic | Couturier et al., 1996; Russell et al., 1996 |
|  | 2001 | 385,000 | 108,000 | Photographic | Couturier et al., 2004 |
|  | 2010 | 74,000 | 13,320 | Photographic | MRNF, 2010 |
|  | 2012 | 27,600 | 2,760 | Photographic | COSEWIC, 2017 |
|  | 2014 | 14,200 | 710 | Photographic | COSEWIC, 2017 |
|  | 2016 | 8,938 | 670 | Photographic | *Québec gov., unpubl. data* |
|  | 2018 | 5,500 | 385 | Photographic | Brodeur et al., 2018 |
|  | 2020 | 8,100 | 486 | Photographic | Brodeur et al., 2021 |
|  | 2022 | 7,200 | 465 | Photographic | *Québec gov., unpubl. data* |

^a^ Aerial photographic surveys were performed by counting caribou present in large groups usually forming during late-June, early-July, when caribou regroup to escape biting flies. Localization of groups was often facilitated by locating individuals equipped with telemetry collars. Observed groups were photographed and all individuals were counted; caribou observed outside of groups and unobserved marked individuals were used to generate the final estimate.

^b^ Visual surveys were performed by counting the number of breeding females on calving grounds or during autumn classifications from the air or the ground. The population size was then extrapolated by dividing the number of observed breeding females by the sex ratio and the proportion of breeding/barren females in the population.

^c^ This estimate was later questioned by Adamczewski et al. (2015). According to these authors, the Beverly herd probably abandoned its traditional range in the mid-1990s to mix with the more abundant Ahiak herd, and thus stopped existing as a distinct herd. The 2011 estimate does not influence our results, however, because morphological data in the Beverly herd were collected between 1980 and 1987.

^d^ Couturier et al. (2004) questioned the validity of the original 2001 estimate and recommended using the lower confidence limit of 628,000 individuals.

^e^ Based on inferences by Boudreau et al. (2003) who studied scars left by caribou hooves on exposed conifer roots in the range of the Rivière-George herd, we set the population peak for this herd in 1988. We used the upper confidence limit of the 1988 population estimate (928,000 individuals) and the lower confidence limit of the 1993 population estimate (719,000 individuals) to generate the population trajectory.

**2. Transforming raw population size estimates to interpolated estimates**

We interpolated population size estimates across all years of the study period. For each herd, we first measured 95% confidence intervals (CI) of survey estimates that did not have one, using the mean observation error calculated on other estimates of the same herd. For the Rivière-aux-Feuilles and Rivière-George herds, we calculated 95% CI from the 90% CI provided in source materials. We fitted locally-weighted polynomial regressions (*loess*) across mean population estimates, as well as across the lower and upper confidence interval bounds of the estimates. For the Rivière-aux-Feuilles and Rivière-George herds, we used additional information from an integrated population model (Vuillaume 2023) to build the *loess* function. We adjusted the span of the *loess* for each herd to ensure the best fit of the smoothing curve to the mean and confidence intervals.

**Table A.1.2.** Estimates of population size for 4 migratory caribou (*Rangifer tarandus*) populations in Canada and Alaska between 1971 and 2010. For each herd the table presents the population estimates based on field surveys (in blue) and the annual population estimates from the locally weighted polynomial regressions (*loess*, in orange), with their 95% confidence intervals in parentheses. For the Rivière-aux-Feuilles and Rivière-George populations, the table also presents the IPM estimates and 95% confidence intervals, based on Vuillaume (2023, in purple).

| **Year** | **Porcupine** | | **Beverly** | | **Rivière-aux-Feuilles** | | | **Rivière-George** | | |
| --- | --- | --- | --- | --- | --- | --- | --- | --- | --- | --- |
|  | **Survey** | ***loess*** | **Survey** | ***loess*** | **Survey** | **IPM** | ***loess*** | **Survey** | **IPM** | ***loess*** |
| 1971 |  |  | **210,000** (141,990–278,010) | **212,785** (143,333–282,238) |  |  |  |  |  |  |
| 1972 | **102,000** (91,417–112,583) | **102,102** (91,508–112,695) |  | **198,229** (134,473–261,984) |  |  |  |  |  |  |
| 1973 |  | **100,859** (90,395–111,324) |  | **184,155** (125,749–242,561) |  |  |  | **100,000** (67,085–132,915) |  | **105,077** (63,654–146,500) |
| 1974 |  | **100,433** (90,013–110,854) | **180,000** (121,705–238,295) | **171,182** (117,449–224,915) |  |  |  |  |  | **152,119** (103,495–200,744) |
| 1975 |  | **100,835** (90,373–111,297) |  | **156,390** (107,748–205,032) | **56,000** (30,027–81,973) |  | **57,660** (31,802–83,518) |  |  | **198,931** (142,113–255,750) |
| 1976 |  | **102,076** (91,486–112,667) |  | **139,437** (96,447–182,427) |  |  | **52,612** (23,982–81,242) | **263,000** (176,433–349,567) |  | **245,432** (179,256–311,609) |
| 1977 | **105,000** (94,106–115,894) | **104,169** (93,361–114,976) |  | **124,808** (86,278–163,339) |  |  | **49,453** (17,841–81,065) |  |  | **292,321** (215,864–368,779) |
| 1978 |  | **107,031** (95,926–118,136) | **130,000** (87,898–172,102) | **116,989** (79,973–154,006) |  |  | **48,391** (13,601–83,179) |  |  | **339,606** (252,012–427,200) |
| 1979 | **110,000** (98,587–121,413) | **110,701** (99,215–122,186) |  | **115,635** (75,663–155,608) |  |  | **49,631** (11,484–87,777) |  |  | **386,168** (286,150–486,186) |
| 1980 |  | **115,535** (103,547–127,522) | **110,000** (74,375–145,625) | **123,073** (77,733–168,414) |  |  | **53,381** (11,711–95,049) | **390,000** (288,723–491,277) |  | **430,891** (316,731–545,051) |
| 1981 |  | **121,480** (108,876–134,085) |  | **153,930** (98,244–209,616) |  |  | **59,847** (14,505–105,188) |  |  | **478,065** (344,662–611,655) |
| 1982 |  | **128,111** (114,819–141,404) | **164,338** (92,006–236,670) | **186,274** (119,853–252,696) |  |  | **69,237** (20,086–118,387) |  |  | **529,351** (371,166–688,113) |
| 1983 | **135,000** (120,993–149,007) | **135,000** (120,993–149,007) |  | **203,750** (131,478–276,022) | **101,000** (49,254–152,746) |  | **81,757** (28,677–134,836) |  |  | **579,683** (395,165–765,130) |
| 1984 |  | **143,307** (128,438–158,176) | **263,691** (183,039–344,343) | **215,884** (139,512–292,256) |  |  | **83,944** (25,706–142,153) | **643,600** (451,770–835,430) |  | **623,996** (415,579–833,409) |
| 1985 |  | **153,095** (137,210–168,979) |  | **220,015** (142,336–297,693) |  |  | **75,105** (10,430–139,691) |  |  | **659,563** (432,137–887,003) |
| 1986 |  | **162,284** (145,446–179,122) |  | **220,614** (143,224–298,003) | **121,000** (54,277–187,723) |  | **74,601** (3,815–145,253) |  |  | **689,489** (449,717–927,200) |
| 1987 | **165,000** (147,880–182,120) | **168,798** (151,284–186,311) |  | **220,867** (143,725–298,009) |  |  | **89,744** (13,343–165,802) |  |  | **717,461** (474,969–955,557) |
| 1988 |  | **173,006** (155,056–190,956) | **189,561** (118,600–260,522) | **223,961** (145,391–302,532) |  |  | **118,221** (36,384–199,280) | **682,100** (388,994–975,206) |  | **747,168** (514,544–973,630) |
| 1989 | **178,000** (159,532–196,468) | **175,589** (157,371–193,807) |  | **229,289** (147,782–310,796) |  |  | **164,957** (78,740–249,961) |  |  | **782,288** (576,195–980,956) |
| 1990 |  | **173,846** (155,809–191,883) |  | **234,646** (149,823–319,469) |  |  | **234,877** (146,210–322,118) |  |  | **817,802** (649,608–977,725) |
| 1991 |  | **168,354** (150,887–185,822) |  | **240,823** (152,109–329,536) | **276,000** (185,566–366,434) |  | **332,907** (244,597–420,023) |  | **962,810** (919,218–986,420) | **958,940** (897,882–1007,259) |
| 1992 | **160,000** (143,399–176,601) | **162,411** (145,560–179,262) |  | **248,609** (155,238–341,980) |  |  | **507,524** (424,144–589,991) |  | **852,229** (805,061–895,924) | **893,215** (831,895–945,233) |
| 1993 |  | **156,695** (140,437–172,954) |  | **258,797** (159,804–357,789) |  |  | **723,565** (648,091–798,783) | **823,000** (699,085–946,915) | **752,317** (721,331–788,711) | **830,286** (769,163–885,152) |
| 1994 | **152,000** (136,229–167,771) | 150,874 (135,220–166,529) | **276,000** (169,400–382,600) | 272,175 (166,404–377,946) |  | **919,560** (853,693–985,528) | 886,274 (822,988–960,127) |  | **885,463** (823,414–937,914) | 770,204 (709,717–827,061) |
| 1995 |  | 145,382 (130,298–160,467) |  | 285,327 (173,236–397,418) |  | **815,181** (746,598–889,026) | 848,597 (783,445–919,730) |  | **652,500** (610,184–696,958) | 713,057 (653,632–771,033) |
| 1996 |  | 139,806 (125,300–154,311) |  | 294,476 (178,181–410,770) |  | **790,178** (729,577–871,171) | 811,148 (744,834–879,390) |  | **658,532** (608,059–708,940) | 658,798 (600,909–717,002) |
| 1997 |  | 134,653 (120,682–148,624) |  | 299,877 (181,366–418,389) |  | **764,539** (706,227–827,953) | 774,102 (707,340–839,250) |  | **628,391** (583,055–673,230) | 607,271 (551,465–664,807) |
| 1998 | **129,000** (115,616–142,384) | 130,434 (116,901–143,967) |  | 301,786 (182,913–420,660) |  | **783,384** (715,452–839,132) | 737,321 (670,829–799,214) |  | **515,357** (482,767–555,422) | 558,326 (505,244–614,082) |
| 1999 |  | 127,002 (113,825–140,178) |  | 300,457 (182,946–417,967) |  | **683,843** (615,318–745,796) | 700,551 (635,040–759,078) |  | **513,811** (472,353–557,991) | 512,032 (462,226–564,945) |
| 2000 |  | 124,306 (111,408–137,203) |  | 296,144 (181,591–410,697) |  | **653,613** (580,994–715,739) | 664,274 (600,263–719,174) |  | **477,699** (443,897–517,408) | 468,563 (422,380–517,964) |
| 2001 | **123,000** (110,238–135,762) | 123,000 (110,238–135,762) |  | 289,102 (178,970–399,234) | **628,000*** *LCI | **613,427** (553,172–653,445) | 628,290 (566,355–679,355) | **385,000** (256,319–513,681) | **408,186** (384,180–435,534) | 428,092 (385,675–473,707) |
| 2002 |  | 123,757 (110,803–136,698) |  | 279,586 (175,208–383,964) |  | **622,517** (566,795–670,211) | 591,317 (532,407–638,697) |  | **427,961** (405,615–453,041) | 394,508 (356,599–435,188) |
| 2003 |  | 126,566 (113,019–140,064) |  | 267,850 (170,429–365,272) |  | **546,094** (495,522–585,675) | 550,674 (498,049–593,248) |  | **377,759** (357,253–402,188) | 366,216 (333,501–400,877) |
| 2004 |  | 131,057 (116,615–145,401) |  | 254,150 (164,757–343,543) |  | **502,410** (453,753–548,724) | 508,156 (463,989–545,148) |  | **325,591** (310,649–340,364) | 335,167 (307,142–364,531) |
| 2005 |  | 136,858 (121,316–152,249) |  | 238,740 (158,316–319,163) |  | **485,606** (436,711–530,257) | 469,206 (430,937–502,617) |  | **282,222** (268,538–296,278) | 298,935 (275,501–323,429) |
| 2006 |  | 143,598 (126,849–160,147) |  | 221,874 (151,230–292,518) |  | **436,859** (401,733–469,127) | 434,266 (397,030–468,052) |  | **290,019** (274,708–305,489) | 261,445 (243,125–280,575) |
| 2007 |  | 150,906 (132,941–168,634) |  | 203,807 (143,623–263,992) |  | **391,739** (361,690–417,533) | 401,267 (362,748–437,638) |  | **236,007** (226,810–245,240) | 224,168 (210,627–238,315) |
| 2008 |  | 158,412 (139,318–177,251) |  | 184,795 (135,620–233,970) |  | **340,657** (318,390–363,670) | 371,884 (332,311–410,272) |  | **213,165** (200,614–225,914) | 188,578 (178,622–198,997) |
| 2009 |  | 165,744 (145,708–185,538) |  | 165,091 (127,344–202,839) |  | **338,835** (311,639–365,041) | 347,390 (307,159–386,990) |  | **175,726** (168,266–183,894) | 152,766 (144,789–161,146) |
| 2010 | **169,000** (153,493–184,403) | 172,531 (151,836–193,033) |  | 144,951 (118,919–170,983) |  | **306,168** (282,949–328,824) | 325,724 (284,931–366,236) | **74,000** (58,129–89,871) | **109,802** (104,828–114,695) | 117,373 (110,557–124,581) |

**BIBLIOGRAPHY**

Adamczewski, J., Gunn, A., Poole, K. G., Hall, A., Nishi, J., & Boulanger, J. (2015). What happened to the Beverly caribou herd after 1994? Arctic, 68(4), 407–421. <http://www.jstor.org/stable/43871357>

Arthur, S. M., Whitten, K. R., Mauer, F. J., & Cooley, D. (2003). Modeling the decline of the Porcupine Caribou Herd, 1989–1998: The importance of survival vs. recruitment. Rangifer, 23(5), 123–130. <https://doi.org/10.7557/2.23.5.1693>

Boudreau, S., Payette, S., Morneau, C., & Couturier, S. (2003). Recent decline of the George River caribou herd as revealed by tree-ring analysis. Arctic Antarctic and Alpine Research, 35(2), 187–195. [https://doi.org/10.1657/1523-0430(2003)035[0187:rdotgr]2.0.co;2](https://doi.org/10.1657/1523-0430(2003)035%5b0187:rdotgr%5d2.0.co;2)

Brodeur, V., Pisapio, J., & McCarthy, S. (2021). Aerial survey of the migratory George River caribou herd in July 2020. Ministère des Forêts, de la Faune et des Parcs, Québec, & Department of Fisheries, Forestry and Agriculture, Newfoundland and Labrador, 21 pp.

Brodeur, V., Rivard, S., Pisapio, J., & McCarthy, S. (2018). Aerial survey of the George River migratory caribou herd in July 2018. Ministère des Forêts, de la Faune et des Parcs, Québec et Department of Fisheries and Land Resources of Newfoundland and Labrador.

Caikoski, J. R. (2020). Porcupine caribou herd management report and plan, Game Management Unit 25A, 25B, 25D, and 26C: Report period 1 July 2012–30 June 2017, and plan period 1 July 2017–30 June 2022. Alaska Department of Fish and Game, Division of Wildlife Conservation, Alaska, 46 pp.

Campbell, M., Boulanger, J., Lee, D. S., Dumond, M., & McPherson, J. (2012). Calving ground abundance estimates of the Beverly and Ahiak subpopulations of barren-ground caribou (*Rangifer tarandus groenlandicus*) – June 2011. Government of Nunavut Department of Environment, 111 pp.

COSEWIC. (2017). COSEWIC assessment and status report on the Caribou *Rangifer tarandus*, Eastern Migratory population and Torngat Mountains population,in Canada. Committee on the Status of Endangered Wildlife in Canada, Ottawa, xvii, 68 pp.

Couturier, S. (1994). Estimation des effectifs du troupeau de caribous de la rivière aux Feuilles en juin 1991, Nord-du-Québec. Ministère du Loisir, de la Chasse et de la Pêche, Québec, 18 pp.

Couturier, S., Courtois, R., Crépeau, H., Rivest, L.-P., & Luttich, S. (1996). Calving photocensus of the Rivière George caribou herd and comparison with an independent census. Rangifer, 16(4), 283–296. <https://doi.org/10.7557/2.16.4.1268>

Couturier, S., Jean, D., Otto, R. D., & Rivard, S. (2004). Demography of the migratory tundra caribou (*Rangifer tarandus*) of the Nord-du-Québec region and Labrador. Ministère des Ressources naturelles, de la Faune et des Parcs, Direction de l’aménagement de la faune du Nord-du-Québec and Direction de la recherche sur la faune, Québec, 68 pp.

Crête, M., Québec (Province). Direction de la gestion des espèces et des habitats, & Québec (Province). Ministère du loisir, de la chasse et de la pêche. (1989). Estimation du nombre de caribous associés à l’aire de mise bas de la rivière George en 1988 et révision des estimations antérieures. Ministère du loisir, de la chasse et de la pêche, Québec, 83 pp.

Crête, M., Le Hénaff, D., Nault, R., Vandal, D., & Lizotte, N. (1987). Estimation du nombre de caribous associés aux aires de mise bas de la rivière aux Feuilles et de la rivière George en 1986. Ministère du Loisir, de la Chasse et de la Pêche, Québec, 31 pp.

Crête, M., Rivest, L.-P., Le Henaff, D., & Luttich, S. N. (1991). Adapting sampling plans to caribou distribution on calving grounds. Rangifer, 11(4), 137–150. <https://doi.org/10.7557/2.11.4.1004>

Fancy, S. G., Whitten, K. R., & Russell, D. E. (1994). Demography of the Porcupine caribou herd, 1983-1992. Canadian Journal of Zoology, 72(5), 840–846. <https://doi.org/10.1139/z94-114>

Goudreault, F., Le Hénaff, D., Crête, M., & Luttich, S. (1985). Dénombrement des caribous sur l’aire de mise bas du troupeau de la rivière George par photographies aériennes verticales en juin 1984. Ministère Du Loisir, de La Chasse et de La Pêche, Québec, Québec, 25 pp.

Gunn, A., & Decker, R. (1982). Survey of the calving grounds of the Beverly herd caribou herd, 1980. Northwest Territories Wildlife Service. Government of the Northwest Territories, File Report No. 20, 27 pp.

Heard, D. (1982). Composition of the Beverly caribou herd in the fall of 1980. Northwest Territories Wildlife Service. Government of the Northwest Territories, File Report No. 26, 11 pp.

Heard, D., & Decker, R. (1980). An estimate of the size and structure of the Beverly caribou herd, 1978–79. Northwest Territories Wildlife Service, File report No. 40, 55 pp.

Heard, D., & Jackson, F. J. (1990). Beverly calving ground survey June 2–14, 1988. Department of renewable resources, Government of the Northwest Territories, Yellowknife, File Report No. 86, 38 pp.

Juniper, I. (1980). Estimé du niveau de population du troupeau de caribous du fleuve George – 1980. Ministère du Tourisme, de la Chasse et de la Pêche, service Québec, 22 pp.

Le Hénaff, D. (1976). Inventaire aérien des terrains de vêlage du caribou dans la région nord et au nord du territoire de la municipalité de la Baie James (mai–juin 1975). Service de la recherche biologique, Ministère du Tourisme, de la Chasse et de la Pêche, Québec, 28 pp.

Le Hénaff, D. (1983). Troupeau de caribous de la rivière aux Feuilles, Nouveau-Québec – Recensement sur le terrain de vêlage. Ministère du Loisir, de la Chasse et de la Pêche, Québec, 14 pp.

Luttich, S. N. (1976). Aerial wintering ground survey of George River caribou concentrations in northern Labrador, February 1975. Newfoundland Labrador Wildlife Division, File report No. 76C-10a, 44 p.

Messier, F., Huot, J., Le Hénaff, D., & Luttich, S. (1988). Demography of the George River caribou herd: Evidence of population regulation by forage exploitation and range expansion. Arctic, 41(4), 279–287. <https://www.jstor.org/stable/40510744>

Ministère des Ressources Naturelles et de la Faune (MRNF). (2010). Communiqué de presse : Résultat de l’inventaire du troupeau de caribous de la rivière George. Chibougamau, le 9 novembre 2010. [Online]. <http://www.mrn.gouv.qc.ca/presse/communiques-detail.jsp?id=8713> [Accessed November 2011]

Pichette, C., & Beauchemin, P. (1973). Inventaire aérien du caribou – février 1973. Ministère du Tourisme, de la Chasse et de la Pêche. Service de la faune. Québec, Québec, 11 pp.

Porcupine Caribou Technical Committee. (2021). Porcupine caribou annual summary report 2020–2021. Porcupine Caribou Management Board, 38 pp. Available from https://pcmb.ca/wp-content/uploads/2021/12/Interim-2021-PCH-Annual-Summary-Report.pdf [Accessed October 12, 2022]

Russell, J., Couturier, S., Sopuck, L. G., & Ovaska, K. (1996). Post-calving photo-census of the Rivière George caribou herd in July 1993. Rangifer, 16(4), 319–330. <https://doi.org/10.7557/2.16.4.1273>

Stephenson, B., Decker, R., & Gunn, A. (1984). Calving ground survey of the Beverly caribou herd, 1982. Northwest Territories Wildlife Service, Yellowknife, NWT, File Report No. 28, 42 pp.

Taillon, J., Brodeur, V., & Rivard, S. (2016). Biological status report of migratory caribou, Leaf River herd. Ministère des Forêts, de la Faune et des Parcs, Québec, 67 pp

Urquhart, D. (1983). The status and life history of the Porcupine caribou herd (1983). Yukon Department of Renewable Resources, Whitehorse, 78 pp.

Williams, T. M. (1995). Beverly calving ground surveys June 5–16 1993 and June 2–13 1994. Department of renewable resources, Government of the Northwest Territories, Yellowknife, File Report No. 114, 46 pp.
