# Supplementary material for: Morphological Traits Are Not Consistently Related to Population Size in Four Migratory Caribou Populations Across North America
Source: Ecol Evol. 2024 Oct 15;14(10):e70468. doi: 10.1002/ece3.70468 (PMC11480523; doi:10.1002/ece3.70468)
Supplement: Supplementary file 2 — Appendix S2. [file ECE3-14-e70468-s003.docx]

**APPENDIX 2:**

**Relationship between morphological traits and date of measurement**

Morphological traits such as body mass and body fat, can considerably vary during a given year, as well as hind foot length of yearlings and even newborns considering the date of birth in June. Because we tried to establish the relationship between these morphological traits with demographic data, we required comparable measures over the years and seasons. We expressed each trait as a linear regression of the date of measurement (in Julian day) testing all increments from 1- to 5-order polynomial (*n*).

$lm ( trait \sim poly \left( date, n \right), data)$ for *n* ≤ 2 and $lm ( trait \sim bs \left( date, df=n \right), data)$ for *n* > 2

We controlled for all model assumptions and used an ANOVA to select the model with the best fit (Pr(>F)). Table A.2.1. presents the polynomial degree retained for each morphological trait, for each age class, at the year and season levels. We then adjusted the morphological trait values to the mean date of measurement at the annual or seasonal scales (Table A3.1) with the best correction we could apply, using the selected linear regression.

**Table A.2.1:** Polynomial order (1 to 5) retained in the best selected linear regression and used to adjust the hind foot length, mass and body fat of each age-sex group to the mean date of measurement. Data were collected on migratory caribou from the Porcupine, Beverly, Rivière-aux-Feuilles (RAF) and Rivière-George (RG) herds, from 1978 to 2010. Seasonal collection periods were originally separated in 3 periods: late winter (Jan‒Apr), summer (May‒Aug), and early winter (Sep‒Dec).

| **Herd** | **Age-sex category** | **Hind foot length** | **Mass** | **Body fat** |
| --- | --- | --- | --- | --- |
| Porcupine | Adult females | Annual: 2 | Annual: 2  Early winter: 1  Late winter: 1 | Early winter: 1  Late winter: 2 |
| Beverly | Adult females | Annual: 3 | Annual: 1  Early winter: 2  Late winter: 1 | Early winter: 1  Late winter: 1 |
| RAF | Adult females | Annual: 2 | Annual: 1  Summer: 1  Early winter: 1 |  |
|  | Yearling females | Annual: 1 | Annual: 1 |  |
|  | Calves | June: 1 | June: 2 |  |
| RG | Adult females | Annual: 3 | Annual: 2  Summer: 2  Early winter: 1  Late winter: 1 | Summer: 1  Early winter: 5  Late winter: 1 |
|  | Yearling females | Annual: 2 | Annual: 1  Summer: 1 |  |
|  | Calves | June: 1 | June: 4 |  |

**Table A.2.2:** Date used to adjust the hind foot length, mass and body fat of each age-sex group to the mean date of measurement. Data were collected on migratory caribou from the Porcupine, Beverly, Rivière-aux-Feuilles (RAF) and Rivière-George (RG) herds, from 1978 to 2010. Only periods during which the relationship between morphological traits and date of measurement were significant are presented in the table. Seasonal collection periods were originally separated in 3 periods: late winter (Jan‒Apr), summer (May‒Aug), and early winter (Sep‒Dec).

| **Herd** | **Age-sex category** | **Hind foot length** | **Mass** | **Body fat** |
| --- | --- | --- | --- | --- |
| Porcupine | Adult females | Annual: 1 Sep. | Annual: 13 Sep.  Early winter: 8 Nov. | Early winter: 10 Nov.  Late winter: 19 Mar. |
| Beverly | Adult females |  | Early winter: 12 Dec. |  |
| RAF | Adult females | Annual: 28 Jul. | Annual: 11 Jun.  Summer: 12 Jun.  Early winter: 27 Oct. |  |
|  | Yearling females | Annual: 12 Jun. |  |  |
|  | Newborns |  | June: 12 Jun. |  |
| RG | Adult females | Annual: 11 Jun. | Annual: 6 Jun.  Summer: 8 Jun.  Late winter: 10 Apr. | Early winter: 23 Oct. |
|  | Yearling females | Annual: 8 Jun. | Annual: 8 Jun.  Summer: 9 Jun. |  |
|  | Newborns | June: 12 Jun. | June: 10 Jun. |  |
