# Supplementary material for: Morphological Traits Are Not Consistently Related to Population Size in Four Migratory Caribou Populations Across North America
Source: Ecol Evol. 2024 Oct 15;14(10):e70468. doi: 10.1002/ece3.70468 (PMC11480523; doi:10.1002/ece3.70468)
Supplement: Supplementary file 3 — Appendix S3. [file ECE3-14-e70468-s001.docx]

**APPENDIX 3:**

**Prior distributions used in Bayesian models**

**Table A.3.1.** Prior distributions used to assess the relationship between morphological traits and population size in 4 migratory caribou (*Rangifer tarandus*) herds in Canada and Alaska: the Porcupine, the Beverly, the Rivière-aux-Feuilles (RAF) and the Rivière-George (RG) herds. These priors were designed based on the empirical data distributions.

| **Herd** | **Age class** | **Sex** | **Morphological trait** | **Parameter** | **Distribution** | **Mean** | **SD** | **LCI** | **UCI** |
| --- | --- | --- | --- | --- | --- | --- | --- | --- | --- |
| Porcupine | Adult | Female | Hind foot length | α | Uniform | – | – | $min(x)$ | $max(x)$ |
|  |  |  |  | β | Uniform | – | – | -1.00 | 1.00 |
|  |  |  |  | σ | Uniform | – | – | 0.00 | 5.00 |
|  |  |  | Mass | α | Uniform | – | – | $min(x)$ | $max(x)$ |
|  |  |  |  | β | Uniform | – | – | -1.00 | 1.00 |
|  |  |  |  | σ | Uniform | – | – | 5.00 | 15.00 |
|  |  |  | Fat | α | Uniform | – | – | $min(x)$ | $max(x)$ |
|  |  |  |  | β | Uniform | – | – | -1.00 | 1.00 |
|  |  |  |  | σ | Uniform | – | – | 0.00 | 5.00 |
| Beverly | Adult | Female | Hind foot length | α | Uniform | – | – | $min(x)$ | $max(x)$ |
|  |  |  |  | β | Uniform | – | – | -1.00 | 1.00 |
|  |  |  |  | σ | Uniform | – | – | 0.00 | 5.00 |
|  |  |  | Mass | α | Uniform | – | – | $min(x)$ | $max(x)$ |
|  |  |  |  | β | Uniform | – | – | 0.00 | 1.00 |
|  |  |  |  | σ | Uniform | – | – | 0.00 | 10.00 |
|  |  |  | Fat | α | Uniform | – | – | $min(x)$ | $max(x)$ |
|  |  |  |  | β | Uniform | – | – | -1.00 | 1.00 |
|  |  |  |  | σ | Uniform | – | – | 0.00 | 5.00 |
| RAF | Adult | Female | Hind foot length | α | Uniform | – | – | $min(x)$ | $max(x)$ |
|  |  |  |  | β | Uniform | – | – | -1.00 | 1.00 |
|  |  |  |  | σ | Uniform | – | – | 0.00 | 5.00 |
|  |  |  | Mass | α | Uniform | – | – | $min(x)$ | $max(x)$ |
|  |  |  |  | β | Uniform | – | – | 0.00 | 1.00 |
|  |  |  |  | σ | Uniform | – | – | 0.00 | 10.00 |
|  | Yearling | Female | Hind foot length | α | Uniform | – | – | $min(x)$ | $max(x)$ |
|  |  |  |  | β | Uniform | – | – | -1.00 | 1.00 |
|  |  |  |  | σ | Uniform | – | – | 0.00 | 5.00 |
|  |  |  | Mass | α | Uniform | – | – | $min(x)$ | $max(x)$ |
|  |  |  |  | β | Uniform | – | – | -1.00 | 1.00 |
|  |  |  |  | σ | Uniform | – | – | 0.00 | 10.00 |
|  | Calf | Male / Female | Hind foot length | α | Uniform | – | – | $min(x)$ | $max(x)$ |
|  |  |  |  | β | Uniform | – | – | -1.00 | 1.00 |
|  |  |  |  | σ | Uniform | – | – | 0.00 | 5.00 |
|  |  |  | Mass | α | Uniform | – | – | $min(x)$ | $max(x)$ |
|  |  |  |  | β | Uniform | – | – | 0.00 | 1.00 |
|  |  |  |  | σ | Uniform | – | – | 0.00 | 5.00 |
| RG | Adult | Female | Hind foot length | α | Uniform | – | – | $min(x)$ | $max(x)$ |
|  |  |  |  | β | Uniform | – | – | -1.00 | 1.00 |
|  |  |  |  | σ | Uniform | – | – | 0.00 | 5.00 |
|  |  |  | Mass | α | Uniform | – | – | $min(x)$ | $max(x)$ |
|  |  |  |  | β | Uniform | – | – | -1.00 | 1.00 |
|  |  |  |  | σ | Uniform | – | – | 0.00 | 15.00 |
|  |  |  | Fat | α | Uniform | – | – | $min(x)$ | $max(x)$ |
|  |  |  |  | β | Uniform | – | – | -1.00 | 1.00 |
|  |  |  |  | σ | Uniform | – | – | 0.00 | 5.00 |
|  | Yearling | Female | Hind foot length | α | Uniform | – | – | $min(x)$ | $max(x)$ |
|  |  |  |  | β | Uniform | – | – | -1.00 | 1.00 |
|  |  |  |  | σ | Uniform | – | – | 0.00 | 5.00 |
|  |  |  | Mass | α | Uniform | – | – | $min(x)$ | $max(x)$ |
|  |  |  |  | β | Uniform | – | – | -1.00 | 1.00 |
|  |  |  |  | σ | Uniform | – | – | 0.00 | 10.00 |
|  | Calf | Male / Female | Hind foot length | α | Uniform | – | – | $min(x)$ | $max(x)$ |
|  |  |  |  | β | Uniform | – | – | -1.00 | 1.00 |
|  |  |  |  | σ | Uniform | – | – | 0.00 | 5.00 |
|  |  |  | Mass | α | Uniform | – | – | $min(x)$ | $max(x)$ |
|  |  |  |  | β | Uniform | – | – | -0.25 | 1.00 |
|  |  |  |  | σ | Uniform | – | – | 0.00 | 5.00 |

$\bar{x}$ = mean of raw data in our sample $SD(x)$ = standard deviation of raw data in our sample

α = intercept of the regression

β = effect of population size on the morphological trait

σ = random effect of individuals

LCI = lower confidence interval from 95% confidence interval

UCI = upper confidence interval from 95% confidence interval
