# Supplementary material for: Morphological Traits Are Not Consistently Related to Population Size in Four Migratory Caribou Populations Across North America
Source: Ecol Evol. 2024 Oct 15;14(10):e70468. doi: 10.1002/ece3.70468 (PMC11480523; doi:10.1002/ece3.70468)
Supplement: Supplementary file 4 — Appendix S4. [file ECE3-14-e70468-s004.docx]

**APPENDIX 4:**

**General descriptive statistics on morphometrics &
Relationship between morphological traits and population size
(seasonal results)**

**General descriptive statistics of morphological trait data**

For all three morphological traits, data distribution ranges (all time period confounded) were highly variable between herds, as well as the inter-annual variability of the traits among herds.

- **Adult females**

For adult females, the average HFL over the study period was the highest for the Rivière-George herd and the lowest for the Beverly. The average body mass over the study period was the highest for the Porcupine herd and the lowest for the Rivière-aux-Feuilles herd. Finally, the average body fat over the study period was the highest for the Beverly herd and the lowest for the Rivière-George herd. Among all herds, inter-annual variability was higher than intra-annual variability for all traits.

**Table A.4.1.** General descriptive statistics on morphometrics of three morphological traits (hind foot length, body mass and body fat) within and across years for adult females of 4 migratory caribou (*Rangifer tarandus*) herds across Canada and Alaska. The table presents the global average over the study period for each trait and herd, the minimum and maximum annual average, the average intra-annual variability (standard deviation) and the inter-annual variability (standard deviation).

| **Morphological trait** | **Herd** | **Global mean** | **Minimum annual average** | **Maximum annual average** | **Average intra-annual variability** | **Inter-annual variability** |
| --- | --- | --- | --- | --- | --- | --- |
| **Hind foot length (cm)** | Porcupine | 40.84 | 39.27 | 43.73 | 1.29 | 2.02 |
|  | Beverly | 37.09 | 36.52 | 38.47 | 1.08 | 1.34 |
|  | Rivière-aux-Feuilles | 55.35 | 54.03 | 55.97 | 1.45 | 1.53 |
|  | Rivière George | 55.77 | 52.93 | 57.47 | 1.85 | 1.90 |
| **Mass (kg)** | Porcupine | 94.70 | 87.41 | 98.99 | 9.28 | 10.05 |
|  | Beverly | 82.69 | 76.83 | 85.80 | 8.24 | 8.30 |
|  | Rivière-aux-Feuilles | 78.81 | 76.92 | 80.74 | 7.59 | 7.85 |
|  | Rivière-George | 86.88 | 80.09 | 92.14 | 9.33 | 10.40 |
| **Fat (index)** | Porcupine | 11.34 | 10.36 | 12.38 | 1.74 | 1.81 |
|  | Beverly | 12.44 | 10.87 | 13.78 | 2.31 | 2.49 |
|  | Rivière-aux-Feuilles | *No data* | | | | |
|  | Rivière-George | 7.03 | 5.22 | 8.41 | 2.98 | 3.65 |

- **Yearlings and calves**

Morphometrics of yearlings and calves were only available for the Rivière-aux-Feuilles and the Rivière-George herds.

Average hind foot length of yearlings over the study period was higher for the Rivière- George herd, while average hind foot length of calves over the study period was higher for the Rivière-aux-Feuilles herd. For yearlings and calves, the average body mass over the study period was higher for the Rivière-George herd. Inter-annual variability was systematically higer than intra-annual variability for all traits.

**Table A.4.2.** General descriptive statistics on morphometrics of three morphological traits (hind foot length, body mass and body fat) within and across years for yearlings and calves of 4 migratory caribou (*Rangifer tarandus*) herds across Canada and Alaska. The table presents the global average over the study period for each trait and herd, the minimum and maximum annual average, the average intra-annual variability (standard deviation) and the inter-annual variability (standard deviation).

| **Morphological trait** | **Herd** | **Global mean** | **Minimum annual average** | **Maximum annual average** | **Average intra-annual variability** | **Inter-annual variability** |
| --- | --- | --- | --- | --- | --- | --- |
| ***Yearlings*** |  |  |  |  |  |  |
| **Hind foot length (cm)** | Rivière-aux-Feuilles | 48.33 | 47.32 | 49.65 | 1.59 | 1.74 |
|  | Rivière-George | 49.61 | 47.15 | 51.71 | 1.63 | 1.99 |
| **Mass (kg)** | Rivière-aux-Feuilles | 39.84 | 36.18 | 45.03 | 4.35 | 4.92 |
|  | Rivière-George | 43.08 | 36.42 | 46.67 | 4.25 | 5.39 |
| **Fat (index)** | Rivière-aux-Feuilles | *No data* | | | | |
|  | Rivière-George | *No data* | | | | |
| ***Calves*** |  |  |  |  |  |  |
| **Hind foot length (cm)** | Rivière-aux-Feuilles | 32.69 | 30.96 | 33.89 | 1.71 | 1.97 |
|  | Rivière-George | 32.64 | 31.65 | 35.53 | 1.73 | 1.97 |
| **Mass (kg)** | Rivière-aux-Feuilles | 5.59 | 5.02 | 6.06 | 0.93 | 0.96 |
|  | Rivière-George | 6.00 | 4.95 | 7.86 | 1.05 | 1.23 |
| **Fat (index)** | Rivière-aux-Feuilles | *No data* | | | | |
|  | Rivière-George | *No data* | | | | |

**Relationships between morphological traits and population size — seasonal results**

**Table A.4.3.** Relationships between estimated population size and mass (kg) in 4 migratory caribou (*Rangifer tarandus*) herds across Canada and Alaska, according to age class and season. Seasonal collection periods were originally separated in 3 periods: late winter (Jan‒Apr), summer (May‒Aug), and early winter (Sep‒Dec). Only years with ≥5 measurements were considered. Rhat is the potential scale reduction factor and an indicator of successful convergence (Rhat < 1.1). n.eff is an estimate of the number of independent samples from the posterior distribution.

| **Herd** | **Age class** | **Sex** | **Morphological trait** | **No. individuals** | **No. years** | **Parameter** | **Mean** | **LCI** | **UCI** | **Rhat** | **n.eff** |  |
| --- | --- | --- | --- | --- | --- | --- | --- | --- | --- | --- | --- | --- |
| Porcupine | Adult | Female | Hind foot length | 189 | 13 | α | 45.70 | 44.76 | 46.09 | 1.00 | 5,000 |  |
|  |  |  |  |  |  | β | **-0.32** | **-0.35** | **-0.26** | 1.00 | 3,300 |  |
|  |  |  |  |  |  | σ | 1.80 | 1.62 | 1.99 | 1.00 | 12,000 |  |
|  |  |  | Mass *(early winter)* | 150 | 10 | α | 76.62 | 71.23 | 86.09 | 1.00 | 9,200 |  |
|  |  |  |  |  |  | β | **0.69** | **0.12** | **0.99** | 1.00 | 6,700 |  |
|  |  |  |  |  |  | σ | 9.70 | 8.89 | 10.61 | 1.00 | 12,000 |  |
|  |  |  | Mass *(late winter)* | 103 | 8 | α | 91.74 | 75.16 | 103.47 | 1.00 | 1,700 |  |
|  |  |  |  |  |  | β | -0.23 | -0.96 | 0.81 | 1.00 | 1,700 |  |
|  |  |  |  |  |  | σ | 9.70 | 8.89 | 10.61 | 1.00 | 12,000 |  |
|  |  |  | Fat  *(early winter)* | 125 | 8 | α | 9.84 | 7.30 | 12.60 | 1.01 | 12,000 |  |
|  |  |  |  |  |  | β | 0.05 | -0.12 | 0.21 | 1.00 | 9,400 |  |
|  |  |  |  |  |  | σ | 1.55 | 1.40 | 1.71 | 1.00 | 12,000 |  |
|  |  |  | Fat  *(late winter)* | 71 | 6 | α | 14.87 | 11.97 | 16.47 | 1.00 | 310 |  |
|  |  |  |  |  |  | β | -0.14 | -0.25 | 0.04 | 1.00 | 320 |  |
|  |  |  |  |  |  | σ | 1.55 | 1.40 | 1.71 | 1.00 | 12,000 |  |
| Beverly | Adult | Female | Hind foot length  *(during population decline)* | 322 | 9 | α | 36.61 | 36.06 | 37.04 | 1.00 | 920 |  |
|  |  |  |  |  |  | β | 0.02 | -0.01 | 0.06 | 1.00 | 790 |  |
|  |  |  |  |  |  | σ | 1.32 | 1.25 | 1.39 | 1.00 | 12,000 |  |
|  |  |  | Hind foot length *(during population increase)* | 327 | 6 | α | 36.61 | 36.06 | 37.04 | 1.00 | 920 |  |
|  |  |  |  |  |  | β | **0.04** | **0.01** | **0.07** | 1.00 | 1,000 |  |
|  |  |  |  |  |  | σ | 1.32 | 1.25 | 1.39 | 1.00 | 12,000 |  |
|  |  |  | Mass *(early winter)* | 191 | 5 | α | 75.03 | 67.65 | 81.01 | 1.00 | 3,600 |  |
|  |  |  |  |  |  | β | **0.31** | **0.04** | **0.65** | 1.00 | 9,200 |  |
|  |  |  |  |  |  | σ | 7.69 | 7.30 | 8.11 | 1.00 | 12,000 |  |
|  |  |  | Mass *(late winter)* | 502 | 8 | α | 68.23 | 62.35 | 73.03 | 1.00 | 2,000 |  |
|  |  |  |  |  |  | β | **0.69** | **0.46** | **0.97** | 1.00 | 1,900 |  |
|  |  |  |  |  |  | σ | 7.69 | 7.30 | 8.11 | 1.00 | 12,000 |  |
|  |  |  | Fat  *(early winter)* | 161 | 5 | α | 11.36 | 8.86 | 13.80 | 1.00 | 1,500 |  |
|  |  |  |  |  |  | β | 0.00 | -0.12 | 0.10 | 1.00 | 1,200 |  |
|  |  |  |  |  |  | σ | 2.31 | 2.19 | 2.45 | 1.00 | 8,200 |  |
|  |  |  | Fat  *(late winter)* | 442 | 8 | α | 9.66 | 7.87 | 10.99 | 1.00 | 4,200 |  |
|  |  |  |  |  |  | β | **0.15** | **0.09** | **0.24** | 1.00 | 3,600 |  |
|  |  |  |  |  |  | σ | 2.31 | 2.19 | 2.45 | 1.00 | 8,200 |  |
| Rivière-aux-Feuilles | Adult | Female | Hind foot length | 197 | 17 | α | 55.27 | 54.26 | 56.31 | 1.00 | 2,400 |  |
|  |  |  |  |  |  | β | 0.00 | -0.01 | 0.02 | 1.00 | 1,900 |  |
|  |  |  |  |  |  | σ | 1.55 | 1.40 | 1.71 | 1.00 | 12,000 |  |
|  |  |  | Mass *(summer)* | 77 | 5 | α | 68.37 | 60.61 | 75.78 | 1.00 | 6,000 |  |
|  |  |  |  |  |  | β | 0.16 | 0.00 | 0.33 | 1.00 | 11,000 |  |
|  |  |  |  |  |  | σ | 7.61 | 6.77 | 8.56 | 1.00 | 9,200 |  |
|  |  |  | Mass *(early winter)* | 71 | 5 | α | 93.97 | 86.07 | 101.62 | 1.00 | 6,800 |  |
|  |  |  |  |  |  | β | -0.05 | -0.122 | 0.12 | 1.00 | 7,600 |  |
|  |  |  |  |  |  | σ | 7.61 | 6.77 | 8.56 | 1.00 | 9,200 |  |
| Rivière-George | Adult | Female | Hind foot length *(during population increase)* | 256 | 13 | α | 55.73 | 55.18 | 56.27 | 1.00 | 5,700 |  |
|  |  |  |  |  |  | β | 0.00 | -0.01 | 0.01 | 1.00 | 5,500 |  |
|  |  |  |  |  |  | σ | 1.91 | 1.79 | 2.04 | 1.00 | 9,400 |  |
|  |  |  | Hind foot length *(during population decline)* | 197 | 16 | α | 55.73 | 55.18 | 56.26 | 1.00 | 5,700 |  |
|  |  |  |  |  |  | β | 0.00 | -0.01 | 0.01 | 1.00 | 5,300 |  |
|  |  |  |  |  |  | σ | 1.91 | 1.79 | 2.04 | 1.00 | 9,400 |  |
|  |  |  | Mass *(summer)* | 102 | 7 | α | 80.26 | 75.98 | 84.39 | 1.00 | 6,500 |  |
|  |  |  |  |  |  | β | 0.01 | -0.10 | 0.12 | 1.00 | 4,900 |  |
|  |  |  |  |  |  | σ | 8.78 | 8.19 | 9.42 | 1.00 | 12,000 |  |
|  |  |  | Mass *(early winter)* | 134 | 9 | α | 101.09 | 97.34 | 104.92 | 1.00 | 3,700 |  |
|  |  |  |  |  |  | β | **-0.10** | **-0.19** | **-0.02** | 1.00 | 3,000 |  |
|  |  |  |  |  |  | σ | 8.78 | 8.19 | 9.42 | 1.00 | 12,000 | |
|  |  |  | Mass *(late winter)* | 171 | 5 | α | 104.62 | 97.807 | 112.28 | 1.00 | 2,900 |  |
|  |  |  |  |  |  | β | **-0.30** | **-0.49** | **-0.14** | 1.00 | 1,800 |  |
|  |  |  |  |  |  | σ | 8.78 | 8.19 | 9.42 | 1.00 | 12,000 |  |
|  |  |  | Fat  *(early winter)* | 110 | 7 | α | 7.93 | 5.41 | 10.87 | 1.00 | 3,400 |  |
|  |  |  |  |  |  | β | -0.02 | -0.07 | 0.03 | 1.00 | 3,200 |  |
|  |  |  |  |  |  | σ | 2.26 | 2.07 | 2.48 | 1.00 | 12,000 |  |
|  | Yearling | Female | Hind foot length | 196 | 18 | α | 49.30 | 48.62 | 49.97 | 1.00 | 12,000 |  |
|  |  |  |  |  |  | β | 0.01 | -0.01 | 0.02 | 1.00 | 12,000 |  |
|  |  |  |  |  |  | σ | 2.00 | 1.81 | 2.21 | 1.00 | 6,600 |  |
|  |  |  | Mass *(summer)* | 134 | 9 | α | 43.55 | 41.91 | 45.13 | 1.00 | 8,400 |  |
|  |  |  |  |  |  | β | -0.02 | -0.06 | 0.02 | 1.00 | 12,000 |  |
|  |  |  |  |  |  | σ | 4.41 | 3.90 | 5.00 | 1.00 | 12,000 |  |

α = intercept of the regression

β = effect of population size on the morphological trait

σ = random effect of individuals

LCI = lower confidence interval from 95% confidence interval

UCI = upper confidence interval from 95% confidence interval
